# The visual pathway in sea spiders (Pycnogonida) displays a simple serial layout with similarities to the median eye pathway in horseshoe crabs

**DOI:** 10.1186/s12915-021-01212-z

**Published:** 2022-01-28

**Authors:** Georg Brenneis

**Affiliations:** Universität Greifswald, Zoologisches Institut und Museum, AG Cytologie und Evolutionsbiologie, Soldmannstraße 23, 17489 Greifswald, Germany

**Keywords:** Evolution, Neuroanatomy, Nervous system, Visual system, Arcuate body, Micro-computed X-ray tomography, Histamine, Tyrosine hydroxylase, Orcokinin, Serotonin

## Abstract

**Background:**

Phylogenomic studies over the past two decades have consolidated the major branches of the arthropod tree of life. However, especially within the Chelicerata (spiders, scorpions, and kin), interrelationships of the constituent taxa remain controversial. While sea spiders (Pycnogonida) are firmly established as sister group of all other extant representatives (Euchelicerata), euchelicerate phylogeny itself is still contested. One key issue concerns the marine horseshoe crabs (Xiphosura), which recent studies recover either as sister group of terrestrial Arachnida or nested within the latter, with significant impact on postulated terrestrialization scenarios and long-standing paradigms of ancestral chelicerate traits. In potential support of a nested placement, previous neuroanatomical studies highlighted similarities in the visual pathway of xiphosurans and some arachnopulmonates (scorpions, whip scorpions, whip spiders). However, contradictory descriptions of the pycnogonid visual system hamper outgroup comparison and thus character polarization.

**Results:**

To advance the understanding of the pycnogonid brain and its sense organs with the aim of elucidating chelicerate visual system evolution, a wide range of families were studied using a combination of micro-computed X-ray tomography, histology, dye tracing, and immunolabeling of tubulin, the neuropil marker synapsin, and several neuroactive substances (including histamine, serotonin, tyrosine hydroxylase, and orcokinin). Contrary to previous descriptions, the visual system displays a serial layout with only one first-order visual neuropil connected to a bilayered arcuate body by catecholaminergic interneurons. Fluorescent dye tracing reveals a previously reported second visual neuropil as the target of axons from the lateral sense organ instead of the eyes.

**Conclusions:**

Ground pattern reconstruction reveals remarkable neuroanatomical stasis in the pycnogonid visual system since the Ordovician or even earlier. Its conserved layout exhibits similarities to the median eye pathway in euchelicerates, especially in xiphosurans, with which pycnogonids share two median eye pairs that differentiate consecutively during development and target one visual neuropil upstream of the arcuate body. Given multiple losses of median and/or lateral eyes in chelicerates, and the tightly linked reduction of visual processing centers, interconnections between median and lateral visual neuropils in xiphosurans and arachnopulmonates are critically discussed, representing a plausible ancestral condition of taxa that have retained both eye types.

**Supplementary Information:**

The online version contains supplementary material available at 10.1186/s12915-021-01212-z.

## Background

In the last two decades, significant advances of phylogenetic analyses leveraging steadily increasing amounts of transcriptomic and genomic data sets have led to the stabilization of the major branches in the arthropod tree of life [1, 2]. However, clarification of the relationships within some of the major lineages is still ongoing and especially the Chelicerata (spiders, scorpions, mites, and their kin) have proved extraordinarily recalcitrant in this respect [3, 4]. While a basal split of the chelicerate lineage into Pycnogonida (sea spiders) and Euchelicerata is now firmly established [5–7], the interrelationships within Euchelicerata are still matter of considerable debate. Recently, one of the most contested issues concerns the position of the marine Xiphosura (horseshoe crabs), which is either placed as sister group of all terrestrial euchelicerates (Arachnida) [7–9] or recovered well-nested within these terrestrial euchelicerate taxa [6, 10–12]. A nested position would have considerable consequences for our understanding of euchelicerate evolution, implying either multiple marine-terrestrial transitions or alternatively a reconquering of marine habitats by horseshoe crabs.

Neuroanatomical studies on Arthropoda have a long history [13, 14]. Already early on, neuroanatomists seeking to resolve the evolutionary relationships of the plethora of disparate taxa have explored the complexity of the central nervous system [15–17], as arthropod diversification was accompanied by pronounced neuroanatomical changes, which serve as a rich substrate for the search of complex shared characters among the different lineages. The last decades have witnessed a resurgence of comparative studies, yielding sophisticated morphological character sets that can be evaluated in the light of opposing phylogenetic hypotheses, contribute to the consolidation of contentious nodes, and shed light on evolutionary transformations of the nervous system over geological time spans [18–26]. Notably, recent studies on the chelicerate visual system have highlighted striking similarities between xiphosurans and several arachnopulmonate taxa (namely scorpions, whip spiders, and whip scorpions) [27–29], which may support the nested placement of horseshoe crabs among terrestrial lineages. However, for character polarization, reliable neuroanatomical data on the brain and its sense organs in the marine sea spiders—the basally branching chelicerate group—are crucial.

Sea spiders are a cosmopolitan but poorly studied component of the benthic fauna in the world’s oceans [30, 31]. Extant representatives have a small body that is divided into an anterior cephalon (or cephalosoma) and the remaining trunk segments, of which the ultimate one bears a small anal tubercle that represents an extremely reduced state of the multi-segmented opisthosoma in other chelicerate taxa [32, 33]. They typically are equipped with four pairs of prominent walking legs (in a few representatives five or six pairs), an anterior proboscis and a dorsal ocular tubercle with two pairs of single-lensed eyes (or ocelli). The structure of the eyes was early on studied with histological methods [34–36] and more recent investigations resolved the fine structure and arrangement of the different cell types, including the photoreceptive retinula cells (R-cells) with a latticed rhabdome [37, 38]. Further, a so-called lateral sense organ (LO), which typically lies between the eyes on the ocular tubercle, has been identified in early studies on some species [36, 39, 40]. Even though fine structural details are available by now, clarification of the LO’s sensory modality is pending [41, 42]. The segmental composition of the pycnogonid brain has been controversially discussed among developmental biologists and neuroanatomists since the late nineteenth century ([34] *vs*. [43, 36] *vs*. [44, 45] *vs*. [46] *vs*. [47]). By now, *Hox* gene expression patterns have helped to settle the debate [48, 49], aligning with neurodevelopmental studies that advocate a bipartite brain comprised of the anterior protocerebral region and the deutocerebral neuromere [43, 47]. Together with the array of separate prosomal ganglia of the ventral nerve cord, this bipartite brain is likely to represent the ancestral state of the chelicerate lineage [50]. However, beyond its gross segmental composition, the neuroanatomy of the pycnogonid brain remains poorly understood. Pioneering work in the early twentieth century revealed surprisingly few structural details [44, 51] and only several decades later, a more comprehensive reconstruction of brain centers and their interconnections was published [52]. Notably, two recent studies expanded and refined available information on the visual system, thereby challenging several of the previous interpretations [38, 53]. They report that each eye is served by two visual neuropils (VNs) that are set up in parallel, i.e., each neuropil is directly targeted by axon terminals of different R-cells from one eye. This contrasts to a serial array of two or three VNs commonly found in euchelicerates and other arthropods [29, 54–56]. Further, Lehmann and colleagues [38] identify a potential homolog of the euchelicerate arcuate body (AB), a higher multimodal integration center that is (among others) thought to be involved in motor control [16, 24, 57, 58]. Characteristically, the euchelicerate AB is a midline-spanning neuropil of crescent shape, subdivided into at least two horizontal layers (or strata) and medio-laterally organized in columnar subunits [16, 21, 24, 59, 60]. While the putative pycnogonid AB is located in proximity to one of the VNs [38], it occupies an untypical central position in the brain, lacks the characteristic shape, and does not display any distinctive horizontal stratification or columnar organization.

Given these unusual findings, the present study sets out to reinvestigate the structure, position, and interconnection of the elements in the pycnogonid visual system, with the additional goal to elucidate for the first time which brain area is targeted by the LO’s sensory afferents [42]. To this end, a multi-methodological approach was chosen, encompassing micro-computed X-ray tomography, histology, fluorescent dye tracing, and immunolabeling of cytoskeletal elements (tubulin) and a marker for synaptic neuropil (synapsin) coupled to 3D analysis and reconstruction. Additionally, immunolabeling of a suite of neuroactive substances (including histamine, tyrosine hydroxylase, 5-hydroxytryptamine, and orcokinin) was performed to explore their distribution patterns and potential for further characterization of sub-regions in the pycnogonid brain. In addition to the fluorescent dye tracing from the eyes, labeling for the biogenic amine histamine was predicted to be particularly helpful for the identification of first-order VNs. Histamine is an inhibitory neurotransmitter utilized by photoreceptive cells across all major arthropod groups [61–68] and thus was expected to highlight the brain centers targeted by histamine-immunoreactive (histamine-ir) R-cell axon terminals. Beyond that, the distribution of tyrosine hydroxylase (TH), the rate-limiting enzyme in the biosynthesis of catecholamines (dopamine, adrenaline, noradrenaline) [69], was a priori considered a promising candidate for further characterization of the VNs and AB and their putative interconnections, as a recent study showed extensive TH-ir synaptic varicosities in these brain centers in spiders [70].

**Fig. 1:**
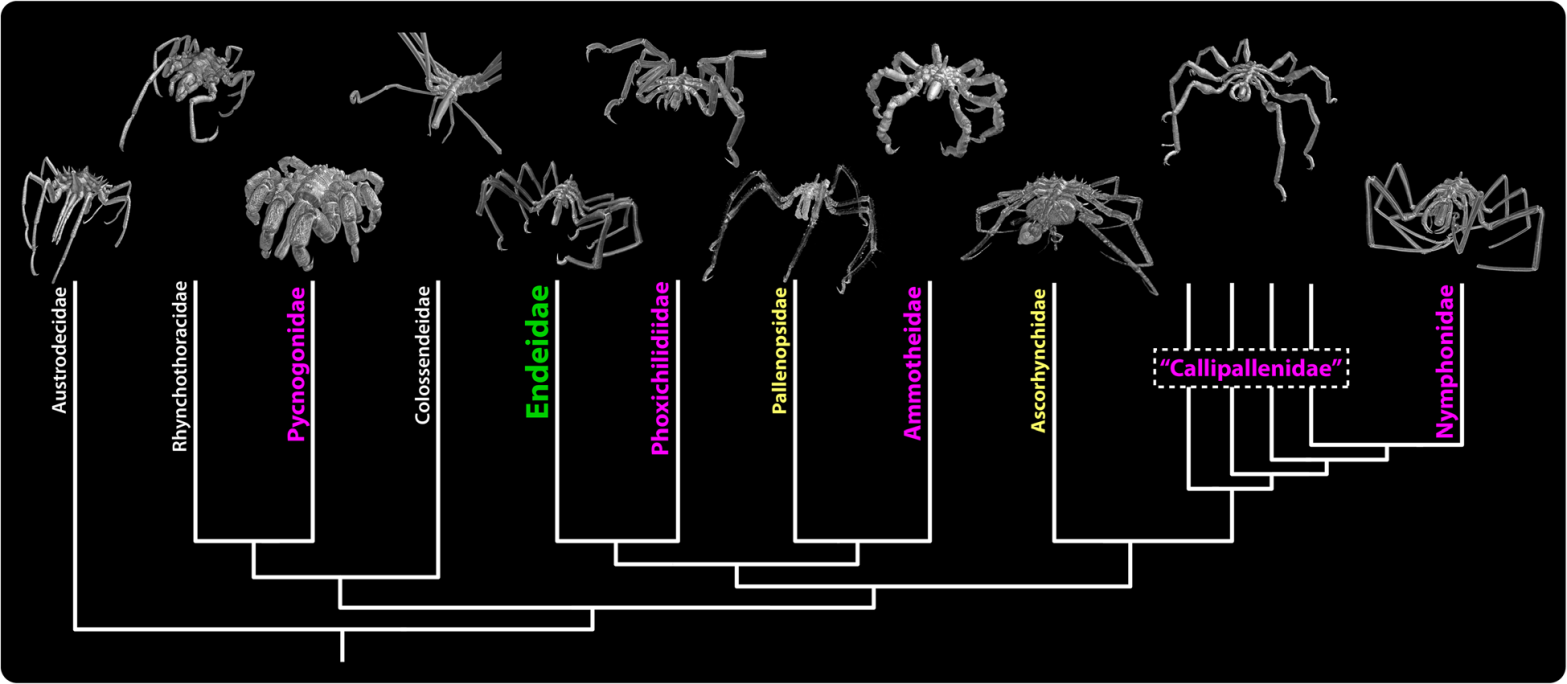
Phylogeny of pycnogonid families and overview of taxa studied. The cladogram is based on [73]. The family Endeidae (green) has been studied with the widest range of methods and highest replication. For comparison, the other families have been investigated in varying detail, the spectrum of methods used and replication decreasing from magenta (Pycnogonidae, Phoxichilidiidae, Ammotheidae, “Callipallenidae”, Nymphonidae) to yellow (Pallenopsidae, Ascorhynchidae) and white (Austrodecidae, Rhynchothoracidae, Colossendeidae). See Additional file 1: Table S1 for more details

To enable direct comparison with previous descriptions, a major focus was put on the species *Endeis spinosa* (Montagu, 1808), which was included in several of the earlier works [35, 38, 42, 52]. As a member of the family Endeidae, *E. spinosa* is positioned well-nested in the pycnogonid tree of life [71–73] (Fig. 1). Beyond that, representatives of all other pycnogonid families were studied in varying detail (Fig. 1; Additional file 1: Table S1), to infer which neuroanatomical features can be traced to the last common ancestor of the pycnogonid crown group, and thus qualify as reliable characters for outgroup comparison. The results on the visual system of Pycnogonida are compared to other chelicerate taxa, and similarities pertaining to the number, developmental sequence, and layout of the adult visual pathway of the median eyes in pycnogonids and xiphosurans are discussed in the light of recent debates on chelicerate phylogeny and evolution.

## Results

The descriptions of brain substructures follow the neuraxis, which deviates from the body axis, as the protocerebrum is turned upward for ca. 90° during development. Accordingly, anterior in the adult neuraxis aligns with dorsal along the body axis, while ventral along the neuraxis represents anterior in terms of body axis, and so forth. If not stated otherwise, images are arranged so that anterior_body axis_ is to the left in sagittal sections/lateral view or to the top in horizontal sections/dorsal view and that dorsal_body axis_ is to the top in cross sections/frontal view.

### Protocerebral sense organs and brain neuroanatomy in *Endeis spinosa* (Endeidae)

#### The anterior and posterior eyes, the LO, and their connection to the brain

**Fig. 2:**
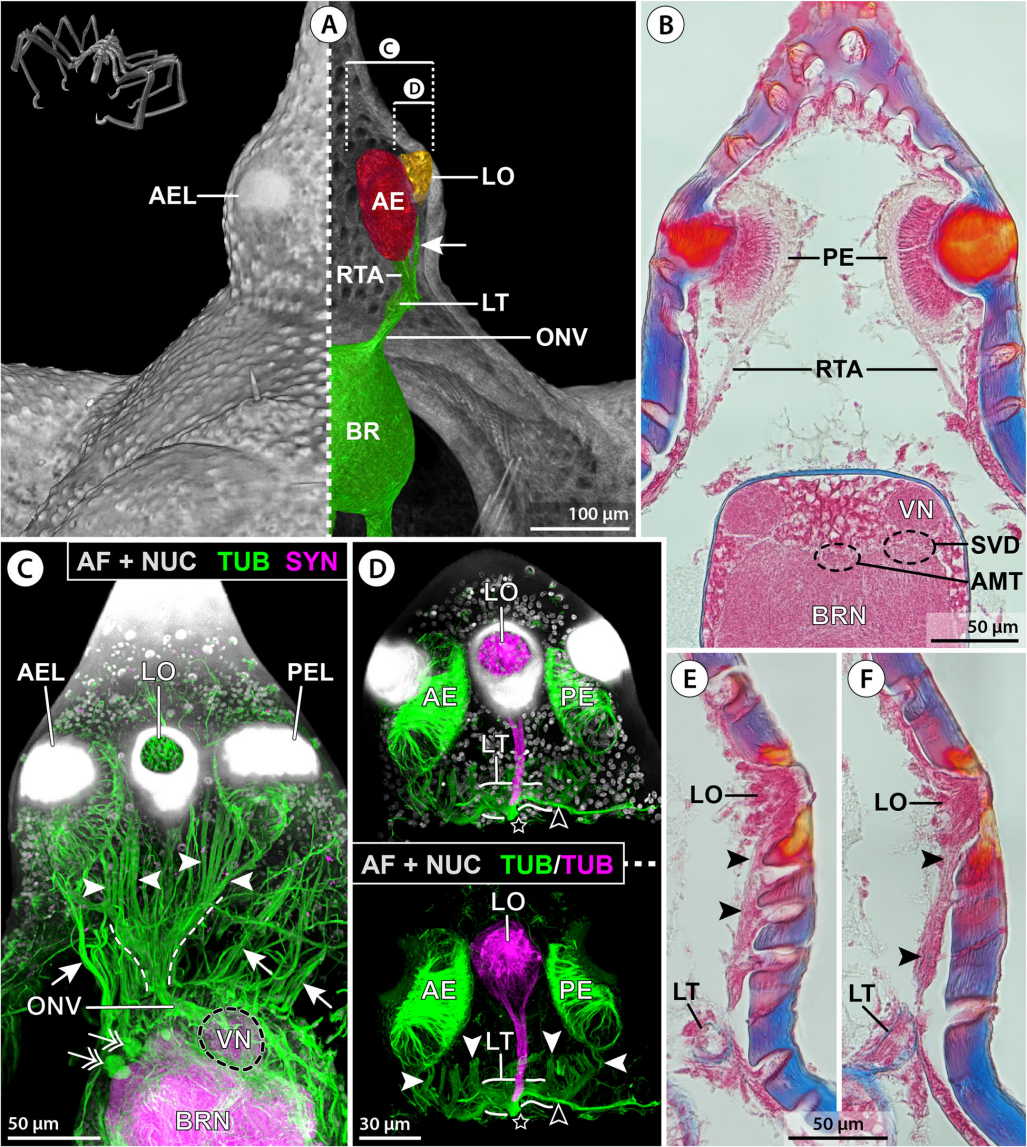
Protocerebral sense organs and their connection to the brain in *E. spinosa*. **A** Micro-CT scan, frontal view. Left half: external view of the ocular tubercle and underlying cephalon region. Right half: 3D-reconstructed volume rendering of the protocerebral sense organs and their nerves to the brain. The arrow points to the lateral sense organ nerve. Stippled brackets indicate the positions of the extended optical sections (MIP) shown in **C** and **D**. **B** Transverse histological section. **C** Tubulin (TUB, green) and synapsin (SYN, magenta) immunolabeling with nuclear counterstain and cuticular autofluorescence (AF + NUC, gray), para-sagittal section. Arrowheads point at selected R-cell axon bundles that successively merge into the lateral thickening (stippled lines). Arrows indicate surrounding strands of connective tissues. Double arrows mark tubulin-rich spherical bodies in the anterior soma cortex. **D** Tubulin immunolabeling (green/magenta) with nuclear counterstain (gray, only in upper image), para-sagittal section. The lateral sense organ and its nerve to the lateral thickening have been segmented and highlighted in a different color. Note that many of the R-cell axon bundles (white arrowheads) have been cut during vibratome sectioning. The black arrowhead points to the nerve connecting to the subepidermal neurite network of the dorsal cephalon (see also Additional file 2: Fig. S1A). The star marks the severed optic nerve extending to the brain. **E,F** Consecutive histological cross sections through the lateral sense organ. Arrowheads point to fibers of the nerve projecting toward the lateral thickening. Abbreviations: AE – anterior eye; AEL – anterior eye lens; AMT – antero-median tract; BR – brain; BRN – brain neuropil; LO – lateral sense organ; LT – lateral thickening; ONV – optic nerve; PE – posterior eye; RTA – R-cell axons; SVD – sub-visual domain; VN – visual neuropil

**Fig. 3:**
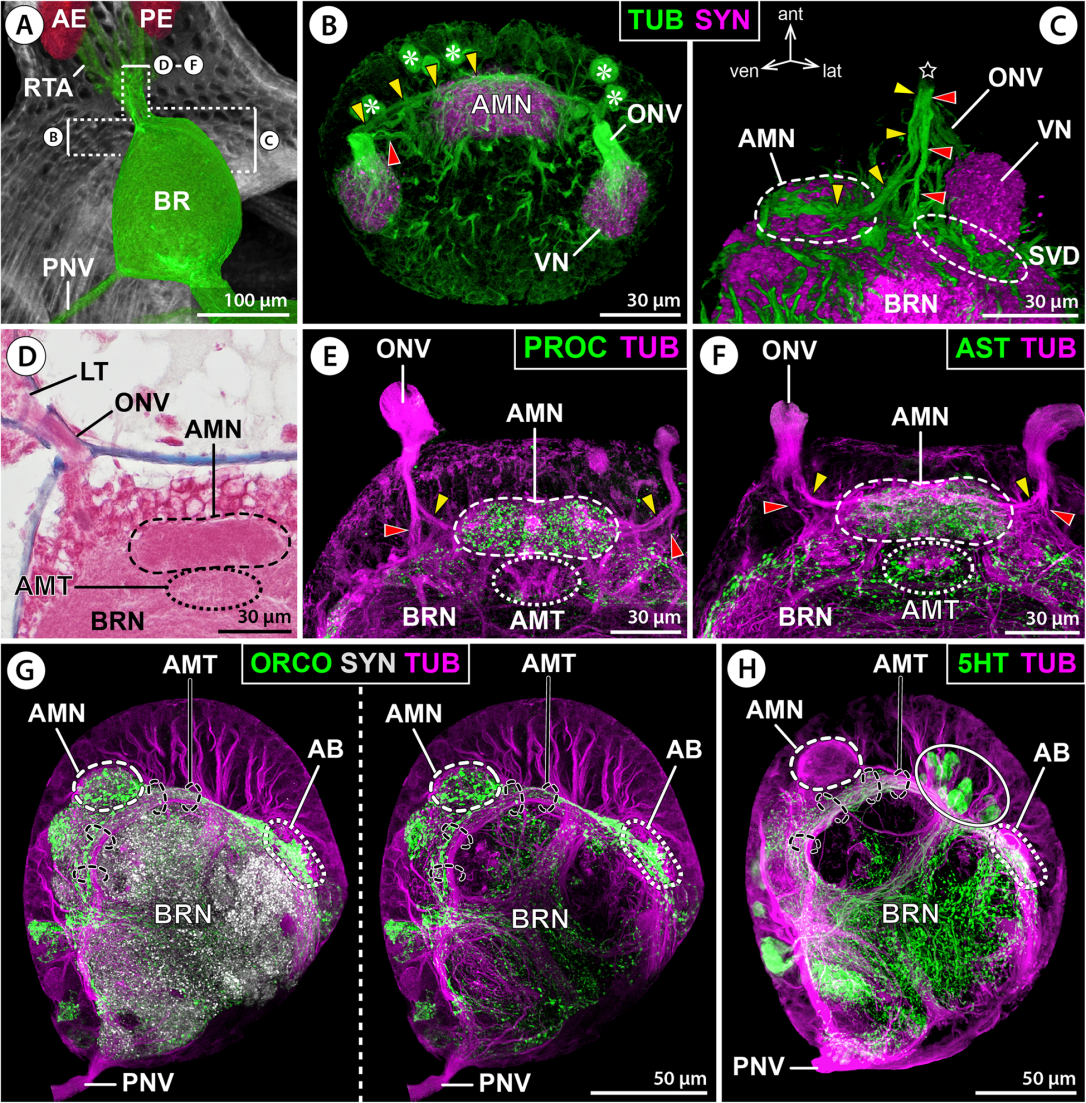
First-order sensory processing centers in the protocerebrum of *E. spinosa*. Immunolabeled samples shown either in MIP (**B**,**E**–**H**) or in blend mode (**C**). Yellow arrowheads mark the optic nerve branch targeting the antero-median neuropil. Red arrowheads point to the optic nerve branch entering the central brain neuropil. **A** 3D-reconstructed volume rendering of part of the eyes and their R-cell axon bundles that merge dorsal to the brain, μCT scan, lateral view. Stippled brackets indicate the positions of the brain areas depicted in **B**–**F**. **B, C** Tubulin (TUB, green) and synapsin (SYN, magenta) immunolabeling. **B** Extended horizontal section. Asterisks mark tubulin-rich spherical bodies in the anterior soma cortex. **C** Oblique lateral view of volume rendering with clipping planes applied. The star indicates the area where the optic nerve enters the soma cortex. **D** Histological cross section. Note the darker staining of the antero-median neuropil, underlain by the less distinct antero-median tract. **E** Tubulin (magenta) and proctolin (PROC, green) immunolabeling, extended cross section. **F** Tubulin (magenta) and allatostatin (AST, green) immunolabeling, extended cross section. **G** Tubulin (magenta), synapsin (gray), and orcokinin (ORCO, green) immunolabeling, extended mid-sagittal section. Synapsin signal omitted in the right image. Note synapsin signal along the antero-median tract, which comprises also some orcokinin-ir neurites to the arcuate body. **H** Tubulin (magenta) and serotonin (5HT, green) immunolabeling, mid-sagittal section. Note serotonin-ir neurites in the antero-median tract. The solid oval highlights an antero-dorsal cluster of serotonin-ir neurons that project their primary neurites directly into the central brain neuropil. Abbreviations: AB – arcuate body; AE – anterior eye; AMN – antero-median neuropil; AMT – antero-median tract; BR – brain; BRN – brain neuropil; LT – lateral thickening; ONV – optic nerve; PE – posterior eye; PNV – proboscis nerve; RTA – R-cell axons; SVD – sub-visual domain; VN – visual neuropil

The ovoid-shaped brain of *E. spinosa* is located in a hemolymph space ventral to the ocular tubercle (Figs. 2A–C and 3A). Numerous tubulin-rich strands of connective tissue extend between the anterior brain surface and the ocular tubercle’s epidermis, anchoring the brain in its position (Fig. 2C). The ocular tubercle bears the paired anterior and posterior eyes and the LO (Fig. 2A,C,D). Each eye is covered by bulbously thickened cuticle (Fig. 2B) that forms a lens and shows strong autofluorescence when excited with UV light (Fig. 2C,D). In agreement with a previous description [38], the axons of the R-cells of each eye project ventrally in several separate bundles (Figs. 2A,B and 3A). These axonal projections successively merge into a thicker bundle that converges with its counterpart of the other ipsilateral eye in a lateral thickening, which is located dorsal to the brain and contains additional neuronal somata (Figs. 2A,C,D and 3D). The LO is located between the anterior and posterior eyes (Fig. 2C,D). It is characterized by a thick cuticle rim that surrounds a circular central area covered only by very thin cuticle layer (Fig. 2C–F). Beneath this area lies a dense cluster of cells, including the sensory cells of the LO. Many (if not all) of the sensory cells feature tubulin-rich processes that converge apically under the thin cuticle cover (Fig. 2C,E,F). Projecting ventrally from the LO, a nerve runs directly beneath the epidermis and merges with the R-cell axon bundles in the lateral thickening (Fig. 2A,D–F). An additional neurite bundle enters the thickening from a postero-lateral direction and converges with the R-cell and LO axons (Fig. 2D). This additional bundle is peripherally connected to a neurite network that spans beneath the epidermis of parts of the ocular tubercle and the antero-dorsal trunk area (Additional file 2: Fig. S1A). From the ventral side of the thickening, one compact optic nerve (ONV) extends to the brain and enters the soma cortex in an antero-lateral position (Figs. 2A,C,F and 3D).

#### First-order sensory processing centers and additional structures in the protocerebrum

After its entry into the brain’s soma cortex, the ONV splits into three branches.

**Fig. 4:**
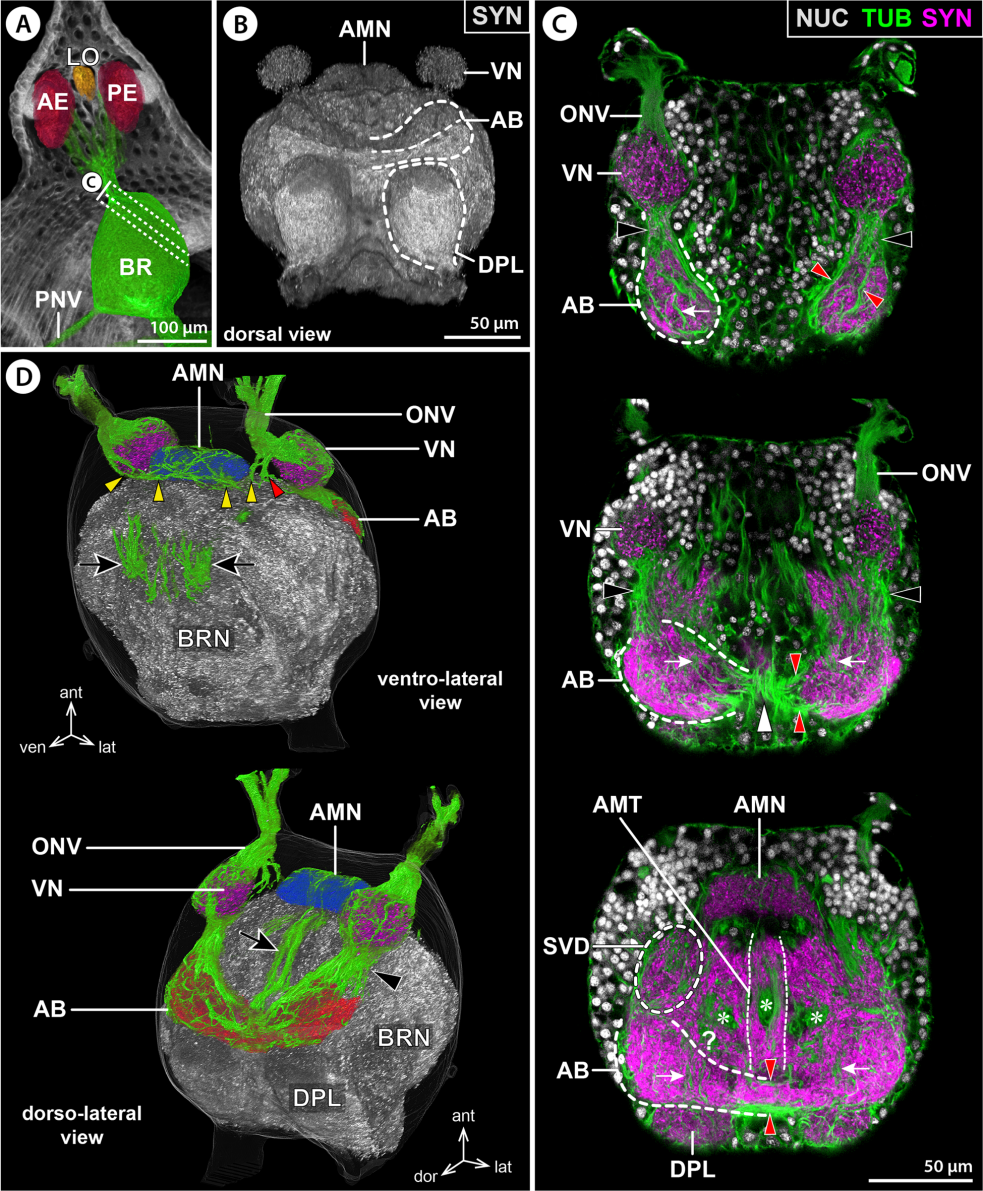
Arcuate body and 3D reconstruction of protocerebral neuropils in *E. spinosa*. **A** 3D-reconstructed volume rendering of protocerebral sense organs and their connection to the brain, μCT scan, lateral view. Stippled lines indicate the positions of the sections shown in **C**. **B** Synapsin (SYN) immunolabeling for neuropil visualization in a whole-mount brain, dorsal view (blend mode). Note different labeling intensities of the two arcuate body layers. **C** Tubulin (TUB, green) and synapsin (magenta) immunolabeling with nuclear counterstain (NUC, gray), oblique optical sections from apical (top) to basal (bottom). Black arrowheads point to the antero-dorsal tract connecting the visual neuropil and arcuate body. Red arrowheads indicate horizontal neurite bundles projecting through the arcuate body. White arrowheads point to selected columnar neurites extending roughly perpendicular to the horizontal bundles. The white arrowhead highlights columnar neurites running on top of the arcuate body’s narrow midline portion. Asterisks mark neurite bundles (cross-sectioned) from antero-dorsal neurons that project into the brain neuropil. **D** 3D volume rendering of a tubulin- and synapsin-labeled brain, ventro-lateral, and dorso-lateral views (top and bottom, respectively). The brain centers were differently colored in the synapsin channel. Only relevant tubulin-labeled nerves/neurite bundles are shown (green). The black arrowhead marks the antero-dorsal tract between the visual neuropil and arcuate body. Yellow arrowheads mark the optic nerve branch targeting the antero-median neuropil. Red arrowheads point to the optic nerve branch entering the central brain neuropil. Black arrows point to the primary neurites of ventral neurons (top) contributing to the antero-median tract (bottom). Abbreviations: AB – arcuate body; AE – anterior eye; AMN – antero-median neuropil; AMT – antero-median tract; BR – brain; BRN – brain neuropil; DPL – dorso-posterior lobe; LO – lateral sense organ; ONV – optic nerve; PE – posterior eye; PNV – proboscis nerve; SVD – sub-visual domain; VN – visual neuropil

(i) The thickest ONV branch extends dorso-posteriorly and innervates the spherical first-order VN that protrudes from the central brain neuropil into the surrounding cortex (Figs. 2B,C, 3B,C, and 4B–D). The VN posteriorly abuds on a region of the central brain neuropil that is here termed sub-visual domain (SVD). The SVD lacks clear morphological borders to the surrounding neuropil and seems to be a hub for passing and arborizing projections, as indicated by tubulin labeling of numerous neurite bundles (Figs. 2B, 3C, and 4C).
(ii) (ii) A more delicate ONV branch extends ventral to the VN, loops medially and merges to the ventral surface of an antero-median neuropil (AMN). The AMN resembles a short, transversally oriented cigar or dumbbell, protrudes likewise into the soma cortex and is in histological sections more intensely stained than the surrounding brain neuropil into which it is posteriorly embedded (Figs. 3B–F and 4B–D). Close to this ONV branch and the AMN, two to five tubulin-rich spherical bodies were found to be embedded in the soma cortex (Figs. 2C and 3B); their nature is currently unclear.
(iii) The third ONV branch projects between the AMN and VN and enters the SVD (Figs. 3B,C,E,F and 4D). It could not be traced any further in tubulin-labeled brains, suggesting that its constituent neurites separate and branch out.

At the brain’s midline, the AMN is underlain by a dome-shaped antero-median tract (AMT). The AMT receives (among others) projections from neurons located in the ventral soma cortex and spans from ventral to dorsal along the surface of the central brain neuropil (Figs. 2B, 3D–H, and 4C,D). Notably, it is not exclusively composed of “naked” axonal projections but displays especially in its ventral portion also neuropilar character, as evidenced by positive synapsin immunolabeling around and between the loosely bundled tubulin-positive neurites (Figs. 2G; 3C). Owing to this neuropilar character and the absence of ensheathing glial cells, the borders of the AMT to the surrounding brain neuropil are not always unequivocally discernible in histological and immunohistological sections (compare, e.g., Figs. 2B and 3D).

The AB resembles a crescent-shaped midline-spanning neuropil that bulges out from the antero-dorsal surface of the central brain neuropil (Fig. 4B,D). In each body half, the tip of the curved AB arm points toward the ipsilateral VN, with which it is connected by a short tract (Fig. 4C,D). Synapsin immunolabeling consistently reveals a horizontal subdivision of the AB into a weakly labeled upper layer and a strongly stained lower layer (Fig. 4B). In contrast to the AB’s clear anterior and posterior borders, its basal side is not as distinctly set off from the central brain neuropil; its extensions are primarily indicated by the course of transverse neurite bundles (Fig. 4C). The AB is antero-dorsally covered by a layer of neuronal somata (Additional file 3: Movie S1). This layer is more prominent near the midline, where the AB narrows and displays an almost commissure-like structure, with condensed transverse neurite bundles and a less prominent neuropil component (Figs. 3G,H and 4B–D). Due to this median narrowing, the AB reveals its underlying bilaterally paired organization. Tubulin immunolabeling shows several slender neurites/neurite bundles that run roughly perpendicular to the transverse neurites, especially in the neuropil-rich lateral AB arms (Fig. 4C). However, an organization into distinct columnar subunits is not apparent. At the midline, the narrow waist of the AB is connected to the dorsally extending AMT (Figs. 3G,H and 4C,D). Posterior to the AB, the dorso-posterior lobe (DPL) protrudes from the central brain neuropil (Fig. 4B–D), flanking the esophagus as it passes the brain.

#### Afferent input into the VN and AMN

**Fig. 5:**
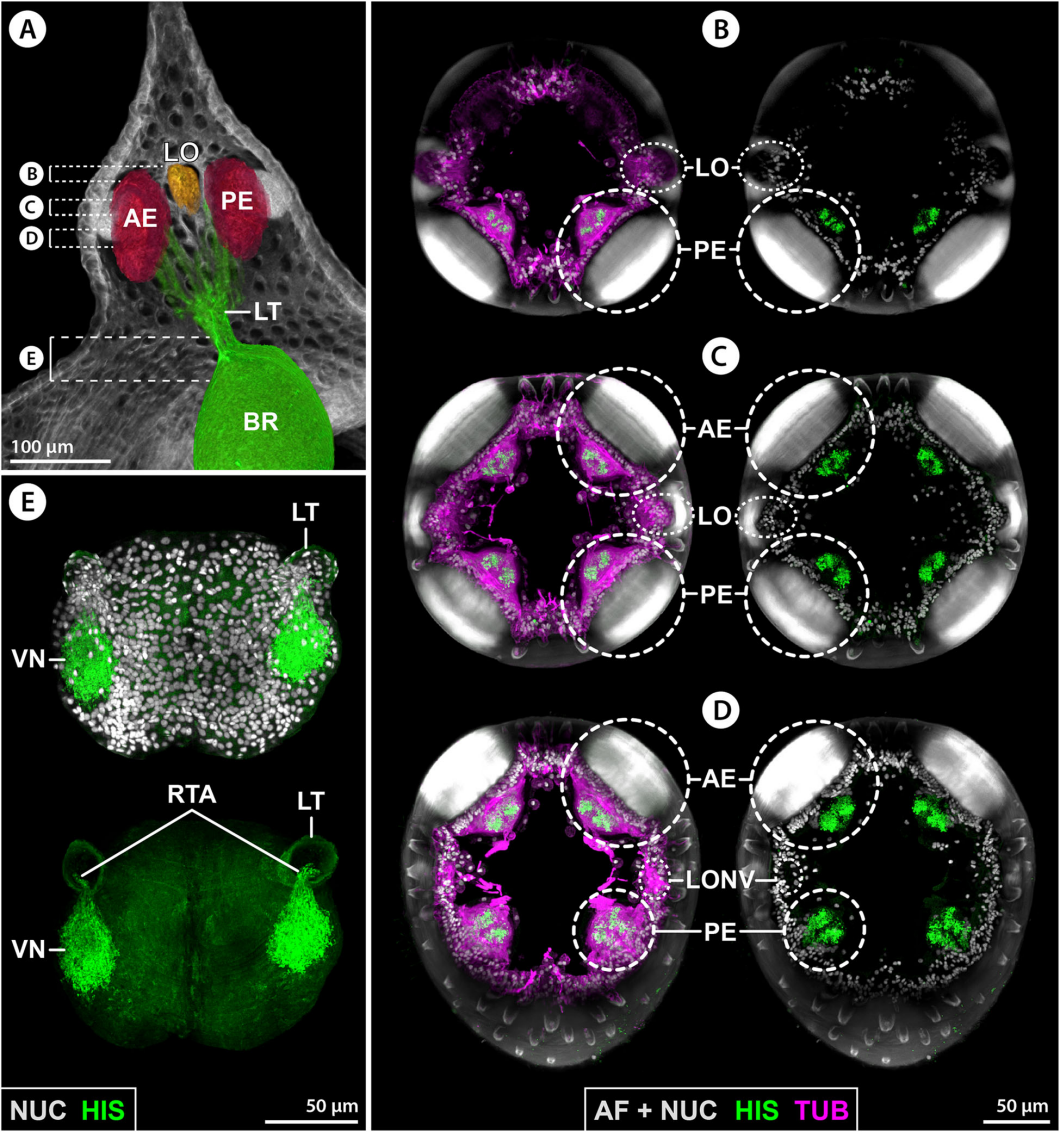
Histamine immunolabeling in eyes and first-order visual neuropils of *E. spinosa.* **A** 3D-reconstructed volume rendering of protocerebral sense organs and their connection to the brain, μCT scan, lateral view. Stippled brackets indicate the positions of the extended sections shown in **B**–**E**. **B–D** Tubulin (TUB, magenta, shown in the left row of images) and histamine (HIS, green) immunolabeling with nuclear counterstain and cuticular autofluorescence (AF + NUC, gray), horizontal sections (MIP) through the ocular tubercle arranged from distal (**B**) to proximal (**D**). Note strong autofluorescence in the cuticular lenses and the cuticle rim of the LO. The R-cells are strongly histamine-ir, while the cells in the LO are devoid of histamine signal. **E** Histamine immunolabeling (green) with nuclear counterstain (gray, only upper image), horizontal section (MIP) through the anterior brain region. Note strong histamine labeling of R-cell axon terminals in the visual neuropils but absence of signal in the median brain region housing the antero-median neuropil. Abbreviations: AE – anterior eye; BR – brain; LO – lateral sense organ; LONV – lateral sense organ nerve; LT – lateral thickening; PE – posterior eye; RTA – R-cell axons; VN – visual neuropil

In a first step toward the characterization of the afferent input into the VN and AMN, immunolabeling for histamine, a neurotransmitter ubiquitously utilized in arthropod photoreceptive cells, was performed. In each eye, the R-cells show strong histamine immunoreactivity along the entire dorso-ventral extension of the retina, whereas none of the cells in the LO are labeled (Fig. 5B–D). Notably, after entering the soma cortex via the lateral thickening and ONV, histamine-ir axons project exclusively along the ONV’s branch toward the ipsilateral VN, where they terminate in a dense synaptic network (Fig. 5E; Additional file 2: Fig. S1B). By contrast, the AMN is devoid of histamine immunolabeling.

**Fig. 6:**
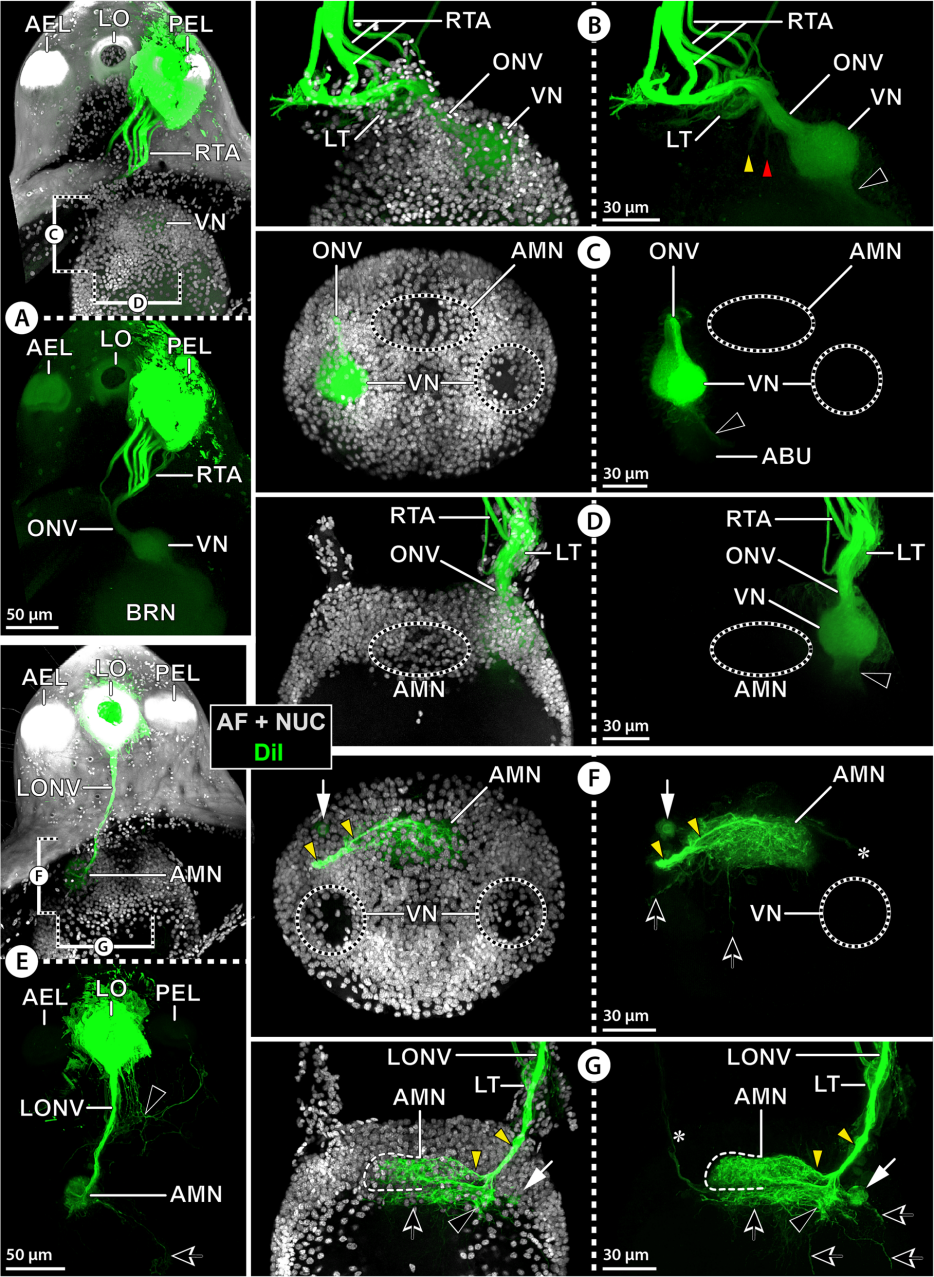
DiI backfills from eyes and lateral sense organ in *E. spinosa.* Extended sections (MIP) of DiI backfills (green) with nuclear counterstain and cuticular autofluorescence (AF + NUC, gray). Counterstain not shown in lower (**A**,**E**) and right (**B**–**D**,**F**,**G**) images. **A**–**D** Backfills from the anterior (**B**–**D**) and posterior (**A**) eyes. **A** Para-sagittal section of ocular tubercle and brain. Note absence of DiI signal in the anterior eye and the lateral sense organ. Stippled brackets indicate the positions of the extended sections shown in **C** and **D**. **B–D** The black arrowhead marks diffuse labeling in the proximal portion of the tract to the arcuate body. The antero-median neuropil is unlabeled. **B** Para-sagittal section. The yellow and red arrowheads point to weak signal in the optic nerve branches to the antero-median neuropil and the central brain neuropil, respectively. **C** Horizontal section. Note stronger labeling in one half of the visual neuropil. **D** Cross section. **E**–**G** Backfills from the lateral sense organ. The visual neuropil is unlabeled. Yellow arrowheads mark neurites in the optic nerve branch projecting to the antero-median neuropil. Black arrows indicate single projections from the antero-median neuropil into the central brain neuropil. White arrows point to a labeled spherical body in the cortex. The asterisk marks a weakly labeled projection toward the contralateral lateral thickening. **E** Para-sagittal section of ocular tubercle and brain. The arrowhead points to neurites of the subepidermal network surrounding the lateral sense organ. Stippled brackets indicate the positions of the extended sections shown in **F** and **G**. **F** Horizontal section. **G** Cross section. Abbreviations: ABU – upper arcuate body layer; AEL – anterior eye lens; AMN – antero-median neuropil; BRN – brain neuropil; LO – lateral sense organ; LONV – lateral sense organ nerve; LT – lateral thickening; ONV – optic nerve; PEL – posterior eye lens; RTA – R-cell axons; VN – visual neuropil

To elucidate the targets of R-cell axon projections with an independent approach, retina backfills (*n*_ae_ = 3; *n*_pe_ = 1) were conducted with the lipophilic marker DiI (Fig. 6). DiI was observed to slowly diffuse across the membranes of neighboring cells with extended incubation times or at higher temperature. As a consequence, increased unspecific labeling of adjacent neural tissue was detectable in the backfills conducted at RT compared to those run at 4 °C. Nonetheless, at the level of resolution aimed for, the results were clear-cut for both temperature settings. From both eyes, DiI-labeled R-cell axon bundles extend toward the thickening and further along the ONV into the ipsilateral VN (Fig. 6A,B,D). Depending on the eye backfilled, one hemisphere within the VN exhibits strong labeling, whereas the other hemisphere shows lower signal intensity (Fig. 6C). Weak marker signal in somata in the thickening, around the VN, and in the proximal portion of the AB tract is likely the result of trans-membrane diffusion of DiI over time (Fig. 6B–D). A similarly diffuse labeling of the proximal portion of the two ONV branches toward the AMN and SVD was observed (Fig. 6B), but the two brain areas themselves do not show any signal (Fig. 6C,D).

In search the origin of afferents targeting the AMN, additional backfills from the LO were performed (*n* = 3). Around the brightly labeled LO, the peripheral network of neurites underlying the ocular tubercle’s epidermis was stained to varying degrees across samples (Fig. 6E). However, the strongest labeling by far is found in the LO nerve axons. They project ventrally, pass through the thickening, extend into the ONV bundle that runs toward the AMN, and branch out lateral to it (Fig. 6E–G). Some axons proceed directly into the AMN and arborize to form dense synaptic varicosities that also penetrate into the AMN’s contralateral side (Fig. 6F,G). Other axons arborize directly posterior to the AMN in the central brain neuropil, with single projections proceeding even deeper in a posterior or dorso-posterior direction (Fig. 6E–G). A weak labeling of single axons in the contralateral ONV branch was found as well (Fig. 6F,G), but whether this is a result of trans-membrane DiI diffusion or indicative of actual axonal projections through the AMN into the contralateral thickening is currently unclear. Further, also some of the tubulin-rich spherical bodies near the labeled axon tract in the anterior soma cortex showed diffuse labeling (Fig. 6F,G). The VN is not targeted by any axonal projections from the LO.

#### Immunolabeling of neuroactive substances in the protocerebral brain centers

To further characterize the protocerebral structures, gain more insights at the cell level, and obtain additional information for subsequent comparison to other pycnogonid species, immunolabeling of different neuroactive substances (in addition to histamine) was conducted.

**Fig. 7:**
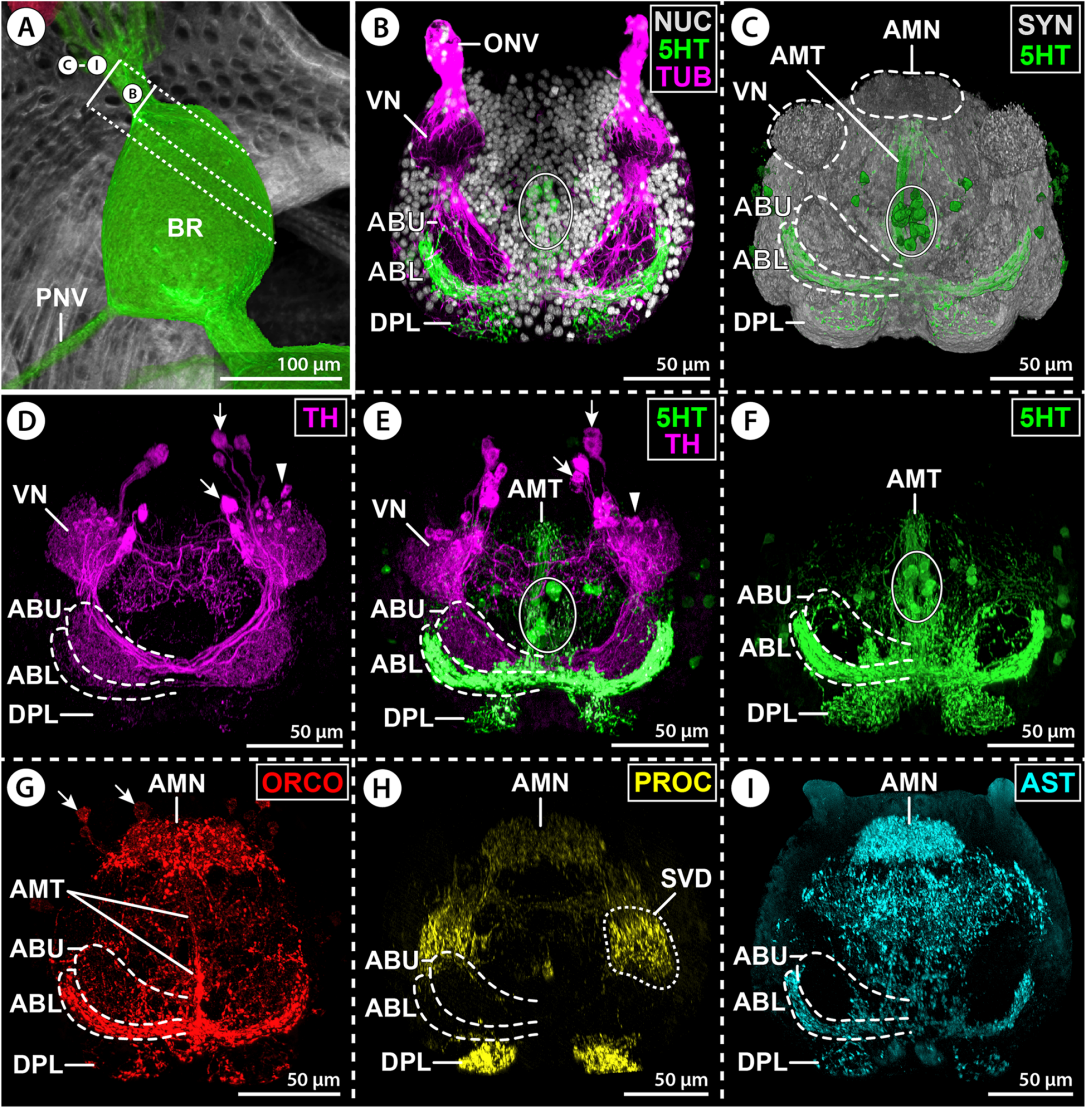
Distribution of neuroactive substances in protocerebral structures of *E. spinosa.* **A** 3D-reconstructed volume rendering of the brain, μCT scan, lateral view. Stippled brackets indicate the positions of the extended sections shown in **B**–**I**. **B**–**I** Oblique sections of immunolabeled whole-mount brains shown either in MIP (**B**,**D**–**I**) or in blend mode (**C**). Solid oval (**B**,**C**,**E**,**F**) demarcates an antero-dorsal cluster of serotonin-ir neuronal somata. **B** Tubulin (TUB, magenta) and serotonin (5HT, green) with nuclear counterstain (NUC, gray). **C** Synapsin (SYN, gray) and serotonin (green). Note absence of serotonin signal in the visual and antero-median neuropils and in the upper arcuate body layer. **D**–**F** Tyrosine hydroxylase (TH, magenta, only **D**,**E**) and serotonin (green, only **E**,**F**). Arrowheads and arrows point at the somata of type 1 and type 2 TH-ir interneurons, respectively. Note largely complementary distribution of TH-ir and serotonin-ir synaptic varicosities in the upper and lower arcuate body layers. **G** Orcokinin (ORCO). Note a few orcokinin-ir projections through the antero-median tract toward the lower arcuate body layer. Arrows point to somata of orcokinin-ir interneurons projecting into the antero-median neuropil. **H** Proctolin (PROC). Note proctolin-ir synaptic varicosities in the antero-median neuropil and sub-visual domain, but lack of signal in the arcuate body. **I** Allatostatin (AST). Note dense allatostatin-ir synaptic network in the antero-median neuropil and the lower arcuate body layer. Abbreviations: ABL – lower arcuate body layer; ABU – upper arcuate body layer; AMN – antero-median neuropil; AMT – antero-median tract; BR – brain; DPL – dorso-posterior lobe; LO – lateral sense organ; LONV – lateral sense organ nerve; LT – lateral thickening; ONV – optic nerve; PNV – proboscis nerve; SVD – sub-visual domain; VN – visual neuropil

The monoamine serotonin (5HT) is strongly labeled in neurons contributing to the AMT and lower AB layer as well as the anterior portion of the DPL (Figs. 3H and 7B,C,E,F; Additional files 3 & 4: Movies S1; S2). The upper AB layer contains only a few serotonin-ir neurites and the VN and AMN are devoid of signal. The AMT receives serotonin-ir neurites from neurons in the ventral soma cortex (Additional file 4: Movie S2). They project dorsally toward the AB, where they merge into the lower layer and give rise to a dense network of synaptic varicosities as they extend tangentially into the tips of its lateral arms (Fig. 7E,F; Additional file 2: Fig. S1E). Some of these neurons seem to send projections further into the DPL (Additional file 2: Fig. S1E). Prior to reaching the AB, the AMT is perpendicularly crossed by a prominent bundle of primary neurites projecting into the central brain neuropil (Figs. 3G,H and 4C). This bundle originates from neurons in the antero-dorsal cortex; several of them display strong serotonin signal and form a conspicuous soma cluster at the midline (Figs. 3H; 7B,C,E,F; Additional file 4: Movie S2).

The enzyme TH is strongly expressed in the VN and the upper AB layer, exhibits only weak labeling in a few neurites in the lower AB layer and DPL, and is absent from the AMN and AMT (Fig. 7D,E; Additional files 3 & 5: Movies S1; S3). Two morphologically different TH-ir interneuron types contribute to the synaptic networks in the VN and AB. Neuron type 1 has a comparably small soma and is arranged in a loose cluster of about ten cells in close vicinity to the VN (Fig. 7D,E; Additional files 2; 5; 6: Figs. S1B; S2C; Movie S3). These neurons arborize in the VN and at least some of them seem to extend a tangential axonal projection through the upper AB layer into the contralateral side. Neuron type 2 has a larger soma and occurs in a loose array of 5–8 cells that are located in the antero-ventral soma cortex. Type 2 neurons feature a distinct primary neurite that extends into the SVD under the VN (Fig. 7D,E; Additional files 5 & 6: Fig. S2C; Movie S3), from where they send prominent axonal projections through the upper AB layer into the contralateral side, but also postero-medially into the central brain neuropil below the AMT (Fig. 7D,E; Additional files 2 & 6: Figs. S1B; S2C). Due to the relatively high number of TH-ir neurons, it could not be resolved (1) whether type 2 neurons also arborize in the ipsilateral VN, (2) if type 1 and/or 2 neurons contribute synaptic varicosities in the ipsilateral AB neuropil, and (3) if the contralateral projections of both neuron types target the VN of the other body half. Notably, in some specimens, TH-ir neurites were found within the ONV (Additional files 2 & 6: Figs. S1B; S2C). The origin of these neurites is unresolved.

The neuropeptide orcokinin (ORCO) is distinctly labeled in the AMN and the lower AB layer, as well as in some neurites in the AMT (Figs. 3G and 7G). Furthermore, scattered synaptic varicosities are found in the upper AB layer and the DPL, whereas the VN lacks signal (Fig. 7G). The somata of several (weakly) orcokinin-ir neurons are located in close vicinity to the AMN (Fig. 7G). Tracing of their primary neurites proved challenging, but a projection toward the AMN could be identified in some cases (Fig. 7G). Some of the orcokinin-ir projections within the AMT originate from neurons in the ventral soma cortex. They contribute to a strongly labeled neuropilar domain ventral to the AMN and potentially also to the AMN itself (Additional file 2: Fig. S1C). After passing through the AMT, their axonal projections merge with the AB, where they branch out tangentially in the lower layer (Fig. 3G, Additional file 2: Fig. S1C,D). Only very few delicate collaterals project in the upper AB layer (Additional file 2: Fig. S1D).

Proctolin as well as allatostatin immunolabeling highlights the AMN (Figs. 3E,F and 7H,I). Similarly, part of the DPL shows immunoreactivity for both neuropeptide classes, whereas the VN is devoid of signal (Fig. 7H,I). While the SVD underlying the VN is only proctolin-ir (Fig. 7H), allatostatin immunolabeling is present in the lower AB layer and in a network of synaptic varicosities in the AMT (Figs. 3F and 7I). The labeling in the different brain areas is consistent for both substances, but signal intensity in neuronal somata and their primary neurites was too weak to draw reliable conclusions regarding the origin of these synaptic networks.

### Protocerebral sense organs and brain neuroanatomy in other pycnogonid families

#### The eyes and R-cell axon bundles

**Fig. 8:**
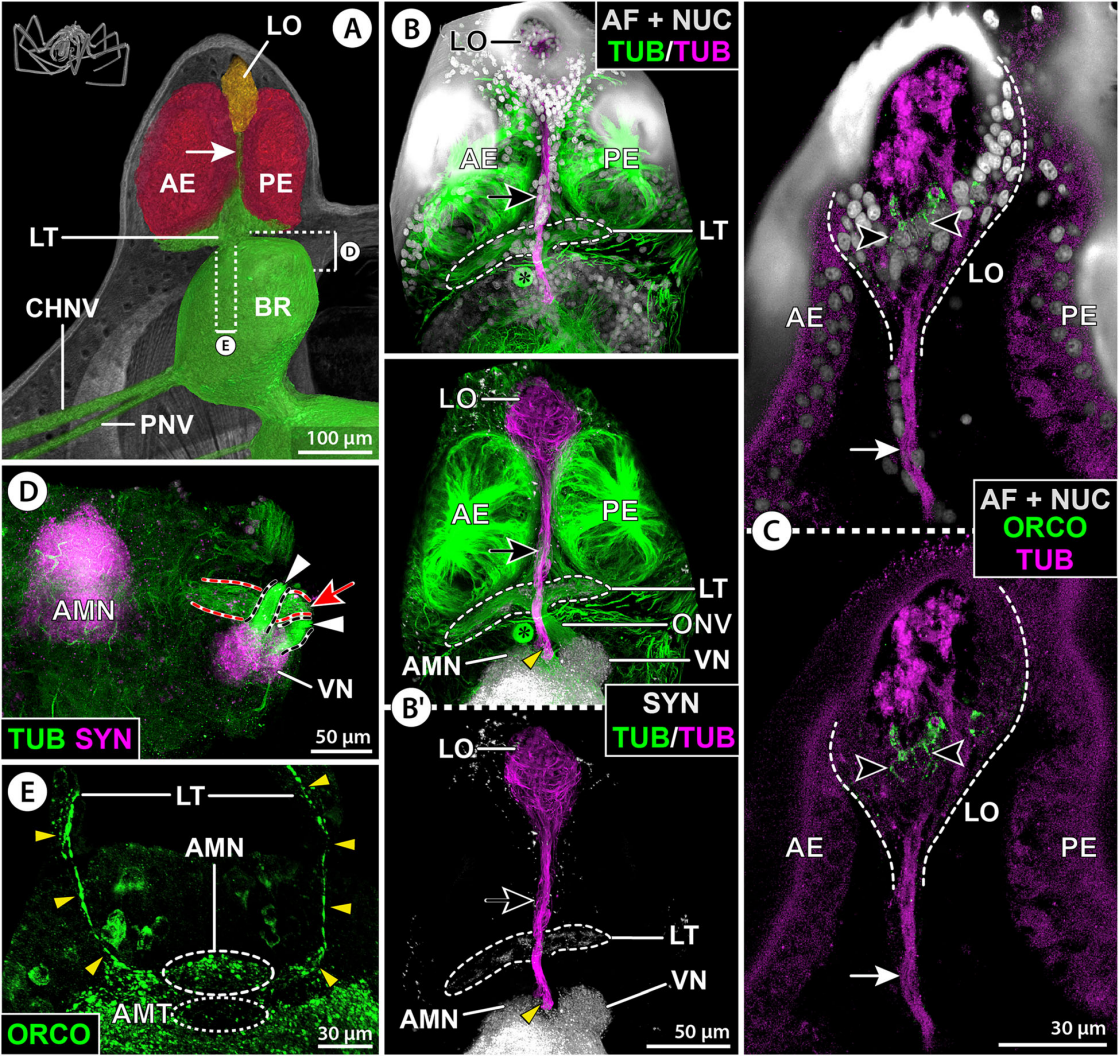
Protocerebral sense organs and their connection to the brain in Nymphonidae. *Nymphon gracile* (**A**–**C**), *N. stroemi* (**D**), and *N.* cf. *multituberculatum* (**E**). **A** 3D-reconstructed volume rendering of protocerebral sense organs and their connection to the brain, μCT scan, lateral view. The arrow points at the lateral sense organ nerve. Stippled brackets indicate the positions of the sections shown in **D** and **E**. **B**–**E** Extended optical sections of immunolabeled samples (MIP). **B**,**B’** Tubulin (TUB, green), para-sagittal section. The tubulin-labeled lateral sense organ and its nerve (arrow) have been segmented and highlighted in magenta. The asterisk marks a tubulin-rich spherical body in the anterior soma cortex. **B** Tubulin with nuclear counterstain and autofluorescence (AF + NUC, gray). **B’** Tubulin with synapsin (SYN, gray), lower image shows only the segmented lateral sense organ with its nerve, which passes through the lateral thickening and diverges from the optic nerve toward the antero-median neuropil (yellow arrowhead). **C** Tubulin (magenta) and orcokinin (ORCO, green) with nuclear counterstain and autofluorescence (gray, only upper image). Arrows mark the lateral sense organ nerve, and arrowheads indicate orcokinin-ir cell bodies in the lateral sense organ. **D** Tubulin (green) and synapsin (magenta), horizontal section. Note bipartition of the axon bundles targeting the visual neuropil (arrowheads) and the separate course of less intensely stained neurites toward the antero-median neuropil (red arrow). **E** Orcokinin, cross section. Single orcokinin-ir neurites pass through the lateral thickening and project toward the antero-median neuropil (yellow arrowheads). Note virtual lack of signal in the poorly defined antero-median tract. Abbreviations: AE – anterior eye; AMN – antero-median neuropil; AMT – antero-median tract; BR – brain; CHNV – cheliphore nerve; LO – lateral sense organ; LT – lateral thickening; ONV – optic nerve; PE – posterior eye; PNV – proboscis nerve; VN – visual neuropil

**Fig. 9:**
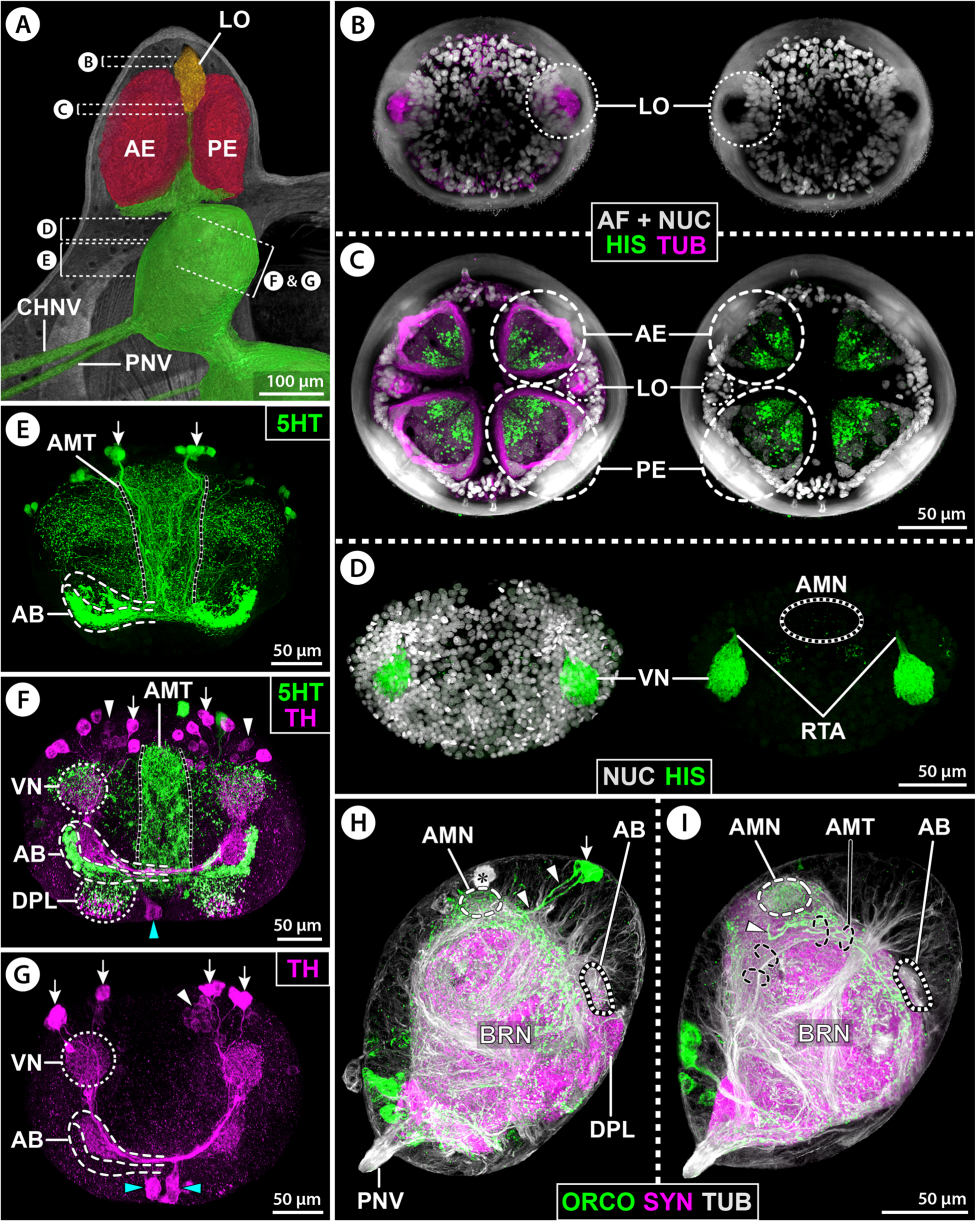
Immunolabeling in sense organs and protocerebral structures in Nymphonidae. *Nymphon gracile* (**A**–**E**,**H**,**I**) and *N.* cf. *multituberculatum* (**F**,**G**). **A** 3D-reconstructed volume rendering of protocerebral sense organs and the brain, μCT scan, lateral view. Stippled brackets indicate the positions of the sections shown in **B**–**G**. **B**–**I** Extended optical sections of immunolabeled samples (MIP). **B**,**C** Tubulin (TUB, magenta) and histamine (HIS, green) with nuclear counterstain and autofluorescence (AF + NUC, gray), horizontal sections. Note strong histamine labeling in the eyes’ retinae (**C**), and absence of signal in the lateral sense organ (**B**,**C**). **D** Histamine (green) with nuclear counterstain (gray), horizontal section. The R-cells send their histamine-ir axon terminals into the visual neuropil. **E** Serotonin (5HT), horizontal section. Arrows indicate somata of selected ventral neurons projecting through the broad antero-median tract to the arcuate body. **F**,**G** Tyrosine hydroxylase (TH, magenta) and serotonin (green, shown only in **F**). Arrows indicate TH-ir somata of selected antero-ventral type 2 neurons. White arrowheads point to selected, weakly TH-ir somata of anterior type 1 neurons. Note complementary TH and serotonin labeling in the arcuate body layers. Light blue arrowheads mark TH-ir somata of dorsal neurons with primary neurites projecting around the arcuate body and extending posteriorly (not shown). **H**,**I** Orcokinin (ORCO, green), synapsin (magenta) and tubulin (gray), para-sagittal and mid-sagittal sections (**H** and **I**, respectively). The arrow indicates orcokinin-ir somata of two anterior neurons with primary neurites (arrowheads) projecting toward the antero-median tract (**H**) before looping into the orcokinin-ir antero-median neuropil (**I**). The asterisk (**H**) marks a tubulin-rich spherical body in the anterior soma cortex. Abbreviations: AB – arcuate body; AE – anterior eye; AMN – antero-median neuropil; AMT – antero-median tract; BR – brain; BRN – brain neuropil; CHNV – cheliphore nerve; DPL – dorso-posterior lobe; LO – lateral sense organ; PE – posterior eye; PNV – proboscis nerve; RTA – R-cell axons; VN – visual neuropil

**Fig. 10:**
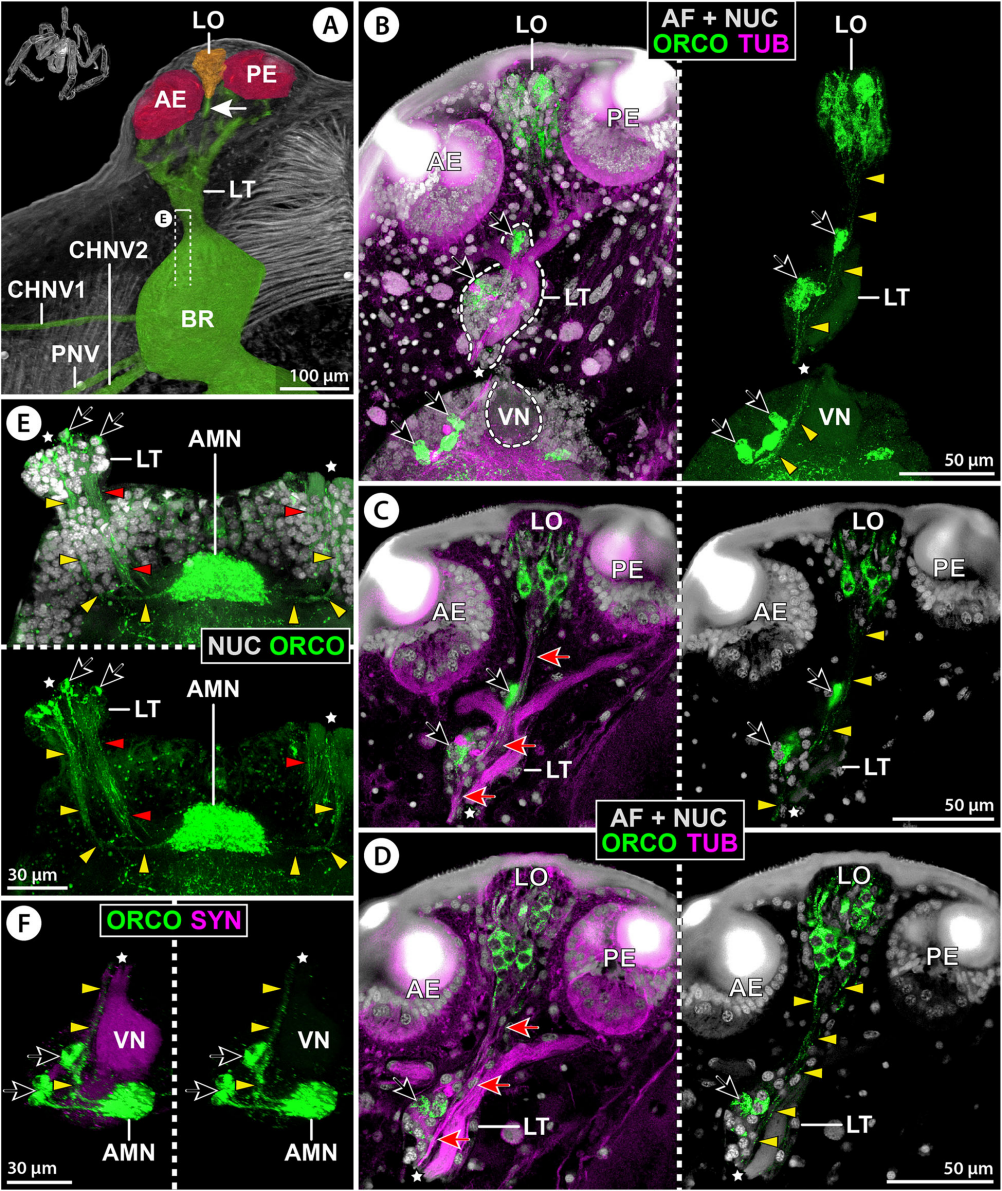
Protocerebral sense organs and their connection to the brain in *Stylopallene cheilorhynchus* (“Callipallenidae”). **A** 3D-reconstructed volume rendering of protocerebral sense organs and the brain, μCT scan, lateral view. The arrow points at the lateral sense organ nerve. The stippled bracket indicates the position of the section shown in **E**. Note that several R-cell axon bundles and the lateral sense organ nerve are slightly damaged. **B**–**F:** Orcokinin (ORCO, green) coupled to tubulin (TUB, magenta; **B**–**D**) or synapsin (SYN, magenta; **F**) immunolabeling with nuclear counterstain and autofluorescence (AF + NUC, gray; left images in **B**–**D**, upper image in **E**). Black arrows mark selected orcokinin-ir somata in the lateral thickening and anterior soma cortex. Red arrows indicate the lateral sense organ nerve. Yellow arrowheads point to thin orcokinin-ir projections of afferents in the lateral sense organ nerve and the optic nerve branch targeting the antero-median neuropil (**E**,**F**). The stars indicate a rupture of the optic nerve at its point of entry into the soma cortex (due to vibratome sectioning). **B** Extended para-sagittal section. Note extensive orcokinin labeling in the lateral sense organ. **C**,**D** Slightly curved, para-sagittal 3D sections. Note finger-shaped apical extensions of the orcokinin-ir sensory cells in the lateral sense organ. **E** Extended cross section. Note the loop of the orcokinin-ir projections into the orcokinin-ir antero-median neuropil and additional projections (red arrowheads) passing the latter. **F** Oblique ventro-lateral view of apical portion of one brain hemisphere. Note absence of orcokinin signal in the visual neuropil and projections of antero-lateral orcokinin-ir neurons into the antero-median neuropil. Abbreviations: AE – anterior eye; AMN – antero-median neuropil; BR – brain; CHNV – cheliphore nerve; LO – lateral sense organ; LT – lateral thickening; PE – posterior eye; PNV – proboscis nerve; VN – visual neuropil

**Fig. 11:**
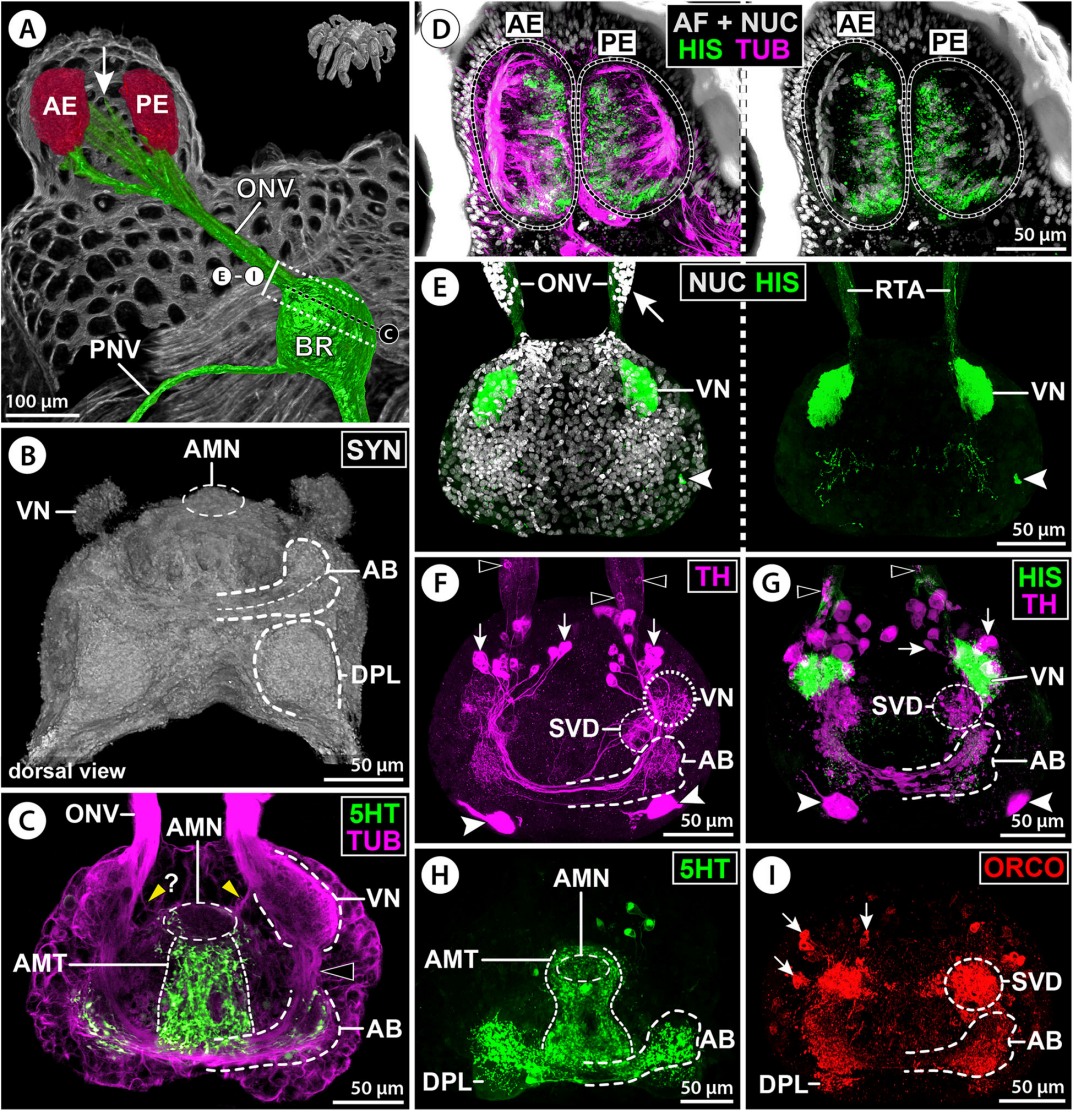
Protocerebral sense organs and their connection to the brain in *Pycnogonum litorale* (Pycnogonidae). **A** 3D-reconstructed volume rendering of protocerebral sense organs and brain, μCT scan, lateral view. A neurite bundle (arrow) extends between the eyes, but the lateral sense organ is lacking. The stippled bracket and black line indicate the positions of (extended) optical sections shown in **C** and **E–I**. **B**–**I** Immunolabeled samples shown in blend mode (**B**) or MIP (**C**–**I**). **B** Synapsin (SYN), whole-mount brain in dorsal view. Note indistinct demarcation of the small antero-median neuropil. **C** Tubulin (TUB, magenta) and serotonin (5HT, green). A neurite bundle (yellow arrowheads) connects the optic nerve and antero-median neuropil. A tract (black arrowhead) connects the visual neuropil and arcuate body. **D** Tubulin (magenta) and histamine (HIS, green) with nuclear counterstain and autofluorescence (AF + NUC, gray), para-sagittal section showing histamine signal in the eyes’ retinae. **E** Histamine (green) with nuclear counterstain. Histamine-ir axon terminals of the R-cells target the visual neuropil. Note somata of the lateral thickening (arrow). The arrowhead marks a neuron projecting posteriorly (not shown). **F**,**G** Tyrosine hydroxylase (TH, magenta) and histamine (green, only in **G**). TH-ir interneurons (arrows) target the visual neuropil and sub-visual domain and project into the arcuate body. Black arrowheads indicate somata in the lateral thickening. White arrowheads highlight somata of posteriorly projecting dorsal neurons (not shown). **H** Serotonin. The antero-median neuropil lies on top of the serotonin-ir antero-median tract. **I** Orcokinin (ORCO). Arrows mark neurons projecting into the sub-visual domain. Note virtual absence of signal in the area housing the antero-median neuropil. Abbreviations: AB – arcuate body; AE – anterior eye; AMN – antero-median neuropil; AMT – antero-median tract; BR – brain; DPL – dorso-posterior lobe; ONV – optic nerve, PE – posterior eye; PNV – proboscis nerve; RTA – R-cell axons; SVD – sub-visual domain, VN – visual neuropil

Micro-CT scans and tubulin immunolabeling in other families reveal a general layout that is very similar to *E. spinosa*. Several R-cell axon bundles project from the anterior and posterior eyes and merge in a thickening prior to entering the brain’s soma cortex in one prominent ONV (Figs. 8A–B’, 10A–D, and 11A; Additional file 7: Fig. S3A). In representatives in which the brain is located at a greater distance to the ocular tubercle, the single axon bundles merge already within/at the base of the ocular tubercle and project from this point on as compact ONV, with neuronal somata being distributed along its length (Fig. 11A;E). Also in the genus *Stylopallene* (“Callipallenidae”), which features a unique subdivision of each eye lens into two parts (Additional file 8: Fig. S4P), each of the bipartite lenses is part of a single internal eye cup with the typical array of emanating axon bundles (Fig. 10A,B). Further, in all families processed for histamine immunolabeling, strong signal was present throughout the eyes’ retina (Figs. 9C and 11D; Additional file 9: Fig. S5A,C,E).

#### The LO and its nerve

In all but one family, the LO could be externally identified in μCT scans of the ocular tubercle, as it shows a characteristic cuticle morphology of a thickened rim surrounding a central area with very thin cuticular cover that often protrudes as a small papilla (Additional file 8: Fig. S4). In several cases, this external identification was further underpinned by μCT study of the internal anatomy and by tubulin immunolabeling (Figs. 8A–C, 9A, and 10A–D; Additional file 7: Fig. S3A). Also in a species lacking functional eyes, the LO is located in its typical position on the ocular tubercle (Additional file 8: Fig. S4D). Only in the family Pycnogonidae, the LO was not found (Fig. 11A; Additional file 8: Fig. S4C).

Projecting ventrally from the LO, a nerve extends into the thickening (Figs. 8B,B’ and 10B–D; Additional file 7: Fig. S3A-C). In some families (Nymphonidae, Phoxichilidiidae; “Callipallenidae”), it could be traced further through the ONV into the soma cortex, where it runs toward the AMN (Figs. 8B,B’,D and 10B–D; Additional file 7: Fig. S3B,C,E). In all families immunolabeled for histamine, the LO cells are devoid of signal (Fig. 9B; Additional file 9: Fig. S5C,E). However, in some species, a subpopulation of the LO cells label for orcokinin (Figs. 8C; 10A–D; Additional file 7: Fig. S3D). For some of these cells, orcokinin-ir projections toward the thin cuticular cover of the LO could be identified, characterizing them as a subset of the LO’s sensory cells (Fig. 10B–D). They send orcokinin-ir axons through the LO nerve, thickening and ONV (Fig. 10B–D; Additional file 7: Fig. S3D).

In correspondence to *E. spinosa*, the presence of an additional nerve that merges into the lateral thickening and connects to a peripheral network of subepidermal neurite bundles of the ocular tubercle and surrounding dorsal cephalon was confirmed for *Phoxichilidium femoratum* (Phoxichilidiidae) (Additional file 7: Fig. S3A).

#### The first-order sensory processing centers VN and AMN

In all families studied, the VN is present. It protrudes into the anterior soma cortex and is targeted by the thickest branch of the ONV (Figs. 8B’,D, 10B, and 11B; Additional files 7 & 10: Figs. S3A-C; S6). As in *E. spinosa*, it receives histamine-ir R-cell axon terminals (Figs. 8D and 11E,G; Additional file 9: Fig. S5B,D,F) and encompasses a TH-ir synaptic network (Figs. 9F,G and 11F; Additional files 6 & 11: Figs. S2; S7A), which in some cases showed only weak signal intensity (e.g., Fig. 11F; Additional file 6: Fig. S2A,D). In some families, single TH-ir neurites were found in the ONV (Additional file 6: Fig. S2A,E) and in one of them (Pycnogonidae) they could be traced to neuronal somata located in the thickening (Fig. 11F,G). Also ventral and antero-ventral clusters of TH-ir interneurons contributing projections to the VN and AB neuropils are reliably present (Figs. 9F,G and 11F,G; Additional file 6: Fig. S2). However, while distinction of type 1 and 2 interneurons was possible in some families (Additional files 6 & 11: Figs. S2B,D-F; S7A), less-pronounced differences in soma sizes and positions impeded unambiguous delimitation in others (e.g., Pycnogonidae; Fig. 11F,G). In contrast to *E. spinosa*, the VN in Nymphonidae and Ammotheidae additionally displays serotonin immunoreactivity (Fig. 9F; Additional file 11: Fig. S7B), but the somata of the affiliated neurons could not be localized.

The AMN was identified in the majority of families studied (Figs. 9H,I, 10E,F, and 11B; Additional file 12: Fig. S8). Only in Austrodecidae, Rhynchothoracidae, and Colossendeidae, its presence could not be satisfactorily clarified, owing to the suboptimal preservation of material available for investigation. The AMN can be more antero-posteriorly flattened than in *E. spinosa*, (e.g., Additional file 12: Fig. S8A,B,D,F), but its characteristic position on top of the AMT and its close association with the ONV branch running ventral to the VN are consistent landmarks (Figs. 9I and 11C,H; Additional files 7 & 12: Figs. S3E; S8). Orcokinin immunolabeling reveals a varicose network in the AMN across families studied, albeit with varying signal intensity (Figs. 8E, 9H,I, and 10E; Additional file 12: Fig. S8D-F). The only exception to this is *P. litorale* (Pycnogonidae), where the small AMN is devoid of signal (Fig. 11I). In the other families, the AMN receives projections from orcokinin-ir interneurons located in the anterior soma cortex (Fig. 9H,I and 10B,F; Additional file 12: Fig. S8D,E). Beyond that, it is targeted by orcokinin-ir neurites via the ventral ONV branch (Figs. 8E and 10E,F; Additional file 12: Fig. S8E,F). In the callipallenid *Stylopallene cheilorhynchus*, these neurites could be traced back to some of the LO’s sensory cells and to a subset of neurons located in the lateral thickening (Fig. 10B–E). In the same species, a second set of orcokinin-ir neurites entering through the ONV projects past the AMN into more posterior brain regions (Fig. 10E).

#### The AMT, AB, and DPL

The AMT is readily identifiable in all families immunolabeled for serotonin, as it consistently encompasses projections from serotonin-ir neurons located in the ventral soma cortex (Figs. 9E,F and 11C,H; Additional files 7 & 13: Figs. S3E; S9). However, the compaction of these neurites and the extent of their synaptic varicosities along the AMT differ between species and families. As a consequence, the AMT represents a rather wide neuropilar band along the midline in some species (e.g., Figs. 9E,F and 11C,H; Additional file 13: Fig. S9C), whereas in others, it is more condensed with distinctive “naked” neurite bundles similar to *E. spinosa* (e.g., Additional file 13: Fig. S9B,D,E,G). Because of this varying degree of neurite condensation in the AMT, its identification in cross sections of synapsin- and tubulin-labeled brains can be challenging (Additional file 12: Fig. S8A-C). Notably, also in the family Austrodecidae, for which the only material available had been stored for several years in PFA fixative, indications for the presence of a serotonin-ir AMT were found (Additional file 13: Fig. S9I). In contrast to the consistently strong serotonin signal in the AMT, orcokinin immunolabeling shows significant interspecific/-familial differences, ranging from strong signal (e.g., Additional file 12: Fig. S8F) to only a few labeled neurites (e.g., Fig. 9C,H; Additional file 12: Fig. S8E) but also to the virtual absence of any labeling (e.g., Fig. 11E; Additional file 12: Fig. S8D).

In all families studied with immunohistological methods, the AB was found in an antero-dorsal position. As in *E. spinosa*, it is divided into two layers, of which the upper one shows immunolabeling for TH and the lower one for serotonin (Figs. 9E–G and 11B,F–H; Additional files 6 & 13: Figs. S2; S9). At the midline, the lower layer is connected to the AMT, from which it receives tangential projections of the serotonin-ir neurons in the ventral soma cortex (Figs. 9E and 11H; Additional files 11 & 13: Figs. S7C; 9). In some species, it displays serotonin-ir varicosities along its entire medio-lateral extension (e.g., Fig. 11H; Additional file 13: Fig. S9A,C,G). In others, serotonin-ir axonal projections are the predominant feature near the midline, thus revealing more distinctly the bilaterally paired organization underlying the AB (e.g., Additional files 11 & 13: Figs. S7C; S9D,E,H). In the upper AB layer, TH-ir synaptic arborizations are concentrated in the lateral arms in all families studied, while the midline is almost exclusively crossed by “naked” axonal projections (Figs. 9F,G and 11F,G; Additional file 6: Fig. S2). As in *E. spinosa*, the tips of the lateral AB arms are connected to the VN and SVD by a tract, along which the antero-ventral and ventral TH-ir neurons project into the upper layer (Figs. 9G; 11C,F; Additional file 6: Fig. S2).

In all families, the DPL is positioned posterior to the AB (e.g., Figs. 9H and 11B). Its anterior portion consistently shows serotonin-ir synaptic varicosities (Fig. 9F and 11H; Additional file 13: Fig. S9).

## Discussion

### Evolutionary conservation of protocerebral sense organs and first-order sensory neuropils

**Fig. 12:**
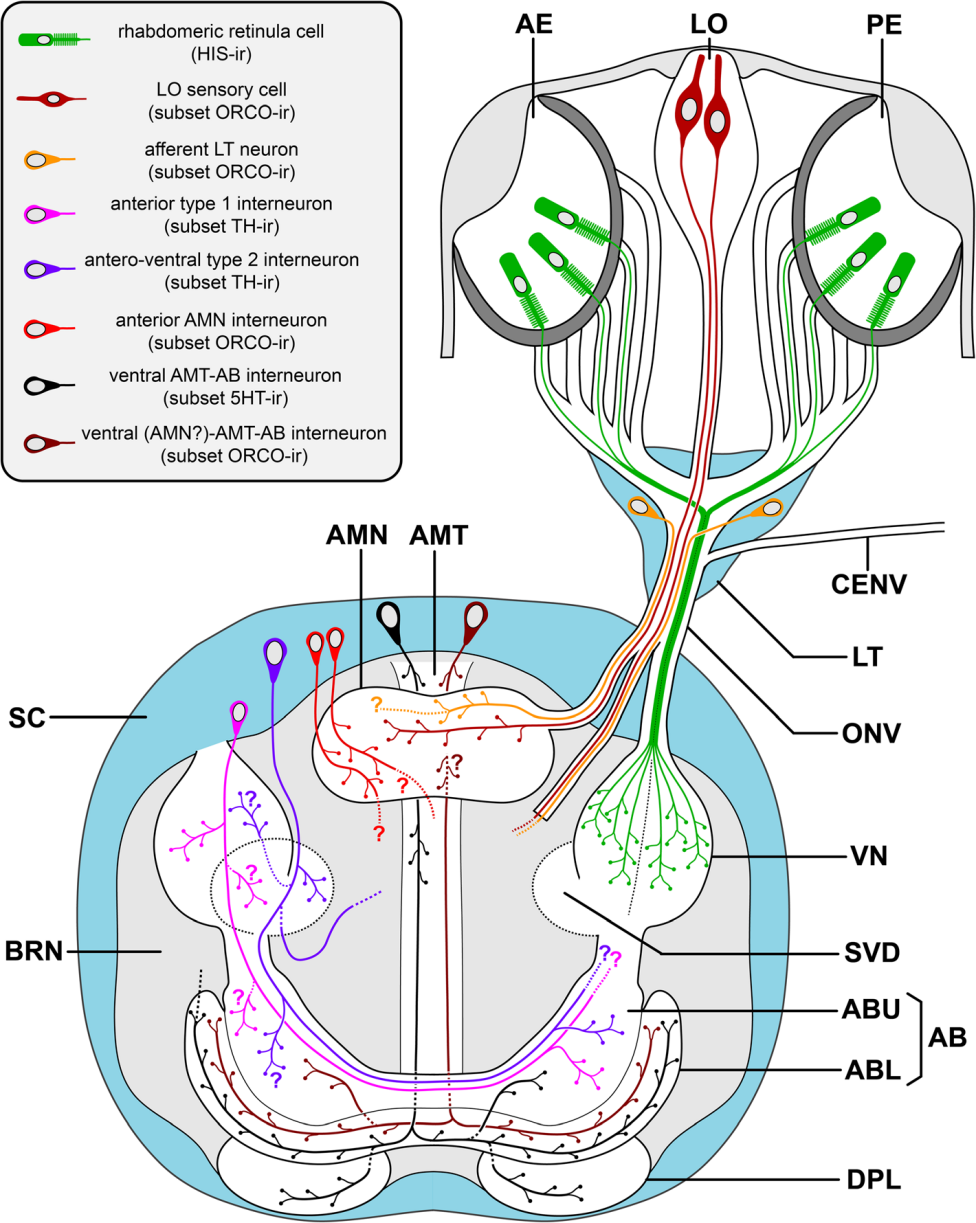
Schematic representation of sense organs, protocerebral centers, and different neuron types in Pycnogonida. The structures depicted are primarily based on results obtained in *E. spinosa* (Endeidae), which in the case of some neuron types have been complemented by findings repeatedly observed in representatives of other families (e.g., the orcokinin-ir sensory cells of the lateral sense organ) and therefore represent a more general feature in Pycnogonida. Question marks and stippled neurite branches mark regions in which projections could not be traced with certainty or were not followed any further posteriorly into the central brain neuropil. Abbreviations: 5HT – serotonin; AB – arcuate body; ABL – lower arcuate body layer; ABU – upper arcuate body layer; AE – anterior eye; AMN – antero-median neuropil; AMT – antero-median tract; BRN – brain neuropil; CENV – (dorsal) cephalon nerve; DPL – dorso-posterior lobe; HIS – histamine; LO – lateral sense organ; LT – lateral thickening; ORCO – orcokinin; ONV – optic nerve, PE – posterior eye; PNV – proboscis nerve; SC – soma cortex; SVD – sub-visual domain, TH – tyrosine hydroxylase; VN – visual neuropil

In this study, *E. spinosa* was studied with the widest range of methods, complementing previous works addressing aspects of this species’ neuroanatomy [35, 38, 42, 52]. Accordingly, the protocerebral sense organs and the sensory processing centers in the protocerebrum are best understood in *E. spinosa* and could be further complemented with findings in several non-endeid representatives (Fig. 12). Comparison with the other families [38, 42, 44, 51–53] shows that the general layout of the sense organs and sensory processing centers is well-conserved. Based on this, many features can be traced back to the last common ancestor of the pycnogonid crown group, including the two pairs of single-lensed eyes and the LO, as well as the VN, AMT, AB, and DPL in the brain (Additional file 14: Table S2). Whether the AMN has been part of the ancestral suite of protocerebral structures remains currently unresolved, as suboptimal sample preservation of the elusive Austrodecidae—the sister group to all other extant families (Fig. 1)—impeded unequivocal clarification. However, given the targeting of the AMN by afferents from the LO in other families, the presence of an equivalent processing center in the austrodecid brain is—by extension—very likely.

Pycnogonidae was found to be the only family lacking the LO. This confirms an older study that did, however, suffer from limited taxon coverage [35]. Owing to the nested position of Pycnogonidae in the sea spider tree of life (Fig. 1), this lack is resolved as a derived condition. Apart from this exception, the comparative analysis highlights an extraordinary evolutionary conservation within Pycnogonida. This is quite remarkable, given that fossil evidence [74] and a recent comprehensive phylogenetic analysis suggest the basal diversification of the pycnogonid crown group to date back to the Ordovician or even earlier [73] and that the sensory equipment and neuroanatomy of the sea spider protocerebrum thus stayed virtually unchanged for more than 425 million years.

### The visual pathway in Pycnogonida revisited: one or two first-order VNs?

Already in histological works, the array of separate axon bundles projecting from each eye and their successive merging in the ipsilateral thickening prior to entry into the brain were discovered [35, 36, 39] and recently confirmed [38]. More discrepancies are found with respect to the number, position, and connectivity of VNs in Pycnogonida [38, 44, 51–53] (Table 1). On the one hand, there is a general consensus regarding the first VN in the soma cortex, which corresponds to the only VN of this study. On the other hand, a second VN has been reported twice. While this putative second neuropil was first suggested to be serially arranged behind the VN [52] (corresponding to the AB of this study), a recent study describes it in a parallel setup to the VN, i.e., as another first-order visual processing center [38] (corresponding to the AMN).

**Table 1:** Comparison of nomenclature of protocerebral pycnogonid brain centers/areas

Hanström [44, 51]	Winter [52]	Lehmann et al. [38, 53]	This study
First-order VN (“Sehmasse”)	First-order VN (“Sehmasse 1”)	First-order VN 1	First-order VN
–	Second-order VN (“Sehmasse 2”)	–	Arcuate body (second-order VN)
–	Corpora pedunculata	First-order VN 2	Antero-median neuropil
–	–	Arcuate body	Antero-median tract (part of)
Corpora pedunculata	“Nebenlappen”	–	Dorso-posterior lobe

In this study, two separate approaches were adopted to identify first-order VNs. In a first step, histamine immunolabeling revealed that the axons of the photoreceptive R-cells terminate exclusively in the VN, but not in the AMN or AB (Fig. 12). In a second step, retinal DiI backfills were performed to independently test for additional targets of potential non-histaminergic R-cells. Since the DiI labeling pattern is congruent with the histamine expression, it indirectly confirms fine structural studies that did not detect R-cell types other than the photoreceptive rhabdomeric one [37, 38]. Accordingly, the VN is resolved as the only first-order visual neuropil in pycnogonids, in line with Hanström [44, 51] and Winter [52] (Table 1).

Interestingly, a subdivision of the VN into two hemi-neuropils has been demonstrated in FIB-SEM reconstructions and each of these hemi-neuropils was proposed to be linked to one of the two eyes in each body half [53]. To some extent this interpretation is supported by differing DiI labeling intensities in two VN hemispheres after backfills from anterior or posterior eyes. For further corroboration, however, simultaneous backfills from both eyes, using different fluorescent tracers would be desirable. Beyond that, fluorescent tracing of subsets of R-cell axon bundles originating from different retina areas would enable to shed light on a potential retinotopic organization of the VN. While an ordered arrangement of the R-cell axon bundles from their origin at the retina to the merging with the lateral thickening has been previously noted [38], direct corroboration of retinotopy in the VN is still missing.

### The elusive arcuate body of Pycnogonida

A brain area designated as AB has been described and depicted only once in Pycnogonida [38]. However, prior to that, two earlier works mention far posterior in the central brain neuropil the so-called central body [44, 52], which was at that time an umbrella term for the unpaired, midline-spanning central body of mandibulate arthropods as well as the AB of chelicerates [15, 57, 75]. Notably, in none of these descriptions does the position, overall shape, and neuroanatomical substructure of the suggested pycnogonid AB show any resemblance to the AB of euchelicerate taxa (for the latter see [16, 57, 60, 75]). In this study, by contrast, the neuropil identified as AB shares the position at the antero-dorsal side of the brain (in terms of neuraxis, i.e., dorso-posterior side in terms of body axis), the crescent shape, and its subdivision into horizontal layers with the AB of xiphosurans and virtually all arachnid taxa studied to date [28, 29, 56, 58, 59, 76–80]. Beyond that, the tip of each lateral arm of the pycnogonid AB is connected to the ipsilateral VN by a tract. More specifically, the upper layer could be shown to receive projections from TH-ir interneurons associated with the VN (see also next section). This conforms with an interpretation of the pycnogonid AB as a higher-order multimodal processing center that integrates (among others) visual cues, in correspondence to the euchelicerate AB [16, 24, 57, 59, 77]. With regard to neuroactive substances other than catecholamines, additional similarities between the pycnogonid and euchelicerate ABs include the absence of significant histamine immunoreactivity [61, 62, 65, 81] and the expression of serotonin in some of its layers [58, 65, 82, 83].

Deviating from the “typical” chelicerate AB, the columnar organization of the pycnogonid AB is not very pronounced and its narrowing close to the midline reveals an underlying bilaterally paired organization. While these features are uncharacteristic for the AB of adult euchelicerates, a look at its development shows interesting parallels. The euchelicerate AB originates from bilaterally paired anlagen that invaginate at the anterior margin of the embryonic head lobes and subsequently assume the characteristic position at the brain’s antero-dorsal side [84–86]. Only in advanced developmental stages, these paired anlagen merge at the midline through ongoing differentiation and integration of additional neurons into its circuits, leading to the unpaired appearance of a prominent midline-spanning AB in adult euchelicerates (e.g., [86]). From a comparative perspective, the AB in adult pycnogonids thus shows some resemblance to an earlier developmental stage of the euchelicerate AB. This less-pronounced AB development and elaboration in pycnogonids may relate to their limited repertoire of complex locomotory patterns. As predators on sessile or slow-moving invertebrates or detritus feeders [87], sea spiders display only sluggish and comparably non-orchestrated limb movements and appear to lack the capacity for the exquisite motor control shown by more active and agile chelicerate representatives. Therefore, a less-pronounced recruitment and integration of neurons into brain centers dedicated to this task may be expected. Since one of the key functions of the chelicerate AB is assumed to be the coordination of motor control of the limbs [16, 24, 57, 58], the lower degree of AB elaboration in pycnogonids fits into this picture.

### Catecholaminergic interneurons relay visual information to the pycnogonid AB

In pycnogonids, details on the downstream processing of the visual information relayed by the R-cells to the VN are very limited. Although a tract extending antero-dorsally from the VN has been reported before, its target region was either not further specified [38], cursorily described as a curved commissure [44] or interpreted as a second-order VN without recognizing its correspondence to the euchelicerate AB [52] (Table 1). In this study, labeling for TH—and thus for putative catecholaminergic neurons [69]—has provided additional insight at the cell level: in all families studied in this regard (Additional file 14: Table S2), clusters of anterior and antero-ventral interneurons form a dense synaptic network in the VN and the underlying SVD. Many—if not all—of them send projections through the antero-dorsal tract into the AB, where their arborizations are predominantly concentrated in the upper layer and also extend into its contralateral side (Fig. 12). This distinct set of catecholaminergic neurons underpins the relay of visual information from the VN to the AB already deduced by the presence of the connecting tract. Interestingly, in euchelicerates, a recent study of TH immunoreactivity in the brain of spiders has revealed a similarly extensive labeling for catecholamines in the VNs and several AB layers [70]. Further, histofluorescent visualization of catecholamines in the brain of xiphosurans has also indicated their presence in neurites of the AB and some VNs [88]. However, in both studies, resolution at the cell level was insufficient to determine whether subsets of interneurons directly connect these brain centers.

Notably, in a FIB-SEM reconstruction of the VN in the pycnogonid *Achelia langi*, Lehmann and colleagues [53] characterized five different types of descending interneurons that are post-synaptic to the R-cell axon terminals, but did not trace them further downstream. In *Achelia echinata*—a close relative of *A. langi*—the soma positions of the anterior TH-ir type 1 neurons directly adjacent to the VN (see Additional file 11: Fig. S7A) agree well with the five described types of descending interneurons. This indicates that at least some of the latter belong to the class of catecholaminergic type 1 neurons projecting to the AB.

The antero-ventral type 2 interneurons do not appear to fall into any of the categories of descending neurons characterized by Lehmann and colleagues [53]. Compared to type 1 neurons, they have more pronounced arborizations in the SVD below the VN (Fig. 12). In addition to projections into the AB, some of them extend branches into the central brain neuropil, underlying the AMN and AMT. This suggests that also additional areas, located in the poorly understood central brain neuropil are involved in the processing of visual cues.

### The AMN is the main target of afferent input from the LO

The LO’s nerve to the lateral thickening was previously documented [36, 44, 89], but its afferents have never been traced into the brain. In *E. spinosa*, DiI backfills from the LO for the first time identify the AMN as main target area of its axons and their synaptic varicosities. This pathway could be further underpinned by orcokinin immunolabeling. In some of the families studied (Additional file 14: Table S2), a subset of orcokinin-ir sensory cells in the LO extend axonal projections through the thickening into the likewise orcokinin-ir AMN (Fig. 12). Based on these two lines of evidence, the AMN is here identified as first-order processing center of the sensory information relayed by the LO. Unfortunately, even though fine structural details are available, the sensory modality of the LO is still unclear, with thermo- or chemoreception being considered the most plausible candidates [41, 42]. An older interpretation of the LO as a rudimentary eye [36] lacks not only support from the cellular fine structure but is likewise challenged by the absence of histamine immunolabeling in its constituent cells.

As mentioned above, the AMN has been previously described as an additional first-order VN set up in parallel to the VN of this study [38] (Table 1). This interpretation is neither supported by the DiI backfills from the eyes of *E. spinosa*, nor by histamine labeling of R-cell axon terminals in any of the pycnogonid families studied here (Additional file 14: Table S2). It is intriguing to note that the pattern of LO axon projections and synaptic varicosities in the AMN as visualized by DiI fills show striking correspondences to the R-cell axon projections into the AMN reported by Lehmann and colleagues [38] (their Fig. 3a, tracing by cobalt fills). Judging by this similarity, one possible explanation for their results is that their documented AMN projections may in fact relate to LO sensory cells that were unintentionally filled. As the eyes and LO lie very close to each other on the narrow ocular tubercle, mislabeling due to unwanted tracer contact with non-target tissues is a feasible source of error for backfills from this region. For this reason, special care was taken in the present study to keep cuticle damage for DiI crystal application as restricted as possible. To additionally control for the specificity of each backfill, the ocular tubercle of each treated specimen was checked for non-target labeling of eyes or LO (see Fig. 6A,E) prior to final brain dissection. Accordingly, even when not taking the independent support from histamine immunolabeling into account, the here described separate pathways of photoreceptive R-cell axons and the LO cell axons are considered reliable.

Intriguingly, the absence of the LO in the family Pycnogonidae does not coincide with an absence of the AMN. Although the latter is relatively small compared to other families and does not display the otherwise characteristic labeling for orcokinin, it is connected with a delicate neurite bundle that branches off the ONV (see Fig. 11C). This may hint toward additional sensory input to the AMN, potentially characterizing it as a multimodal processing center in other pycnogonid families. One plausible source of additional sensory input could be afferents from the peripheral subepidermal neurite network connected to the lateral thickening, as here described for *E. spinosa* and *Ph. femoratum*. Future studies using backfills from setae on the ocular tubercle and cephalosoma should aim to explore this issue further.

It is interesting to note that Winter [52] claimed the AMN to represent the “corpora pedunculata” (Table 1), i.e., considered them to be part of the mushroom bodies, a higher-order multimodal sensory integration center in the protocerebrum of chelicerates and other arthropod lineages [16, 24, 26, 90]. However, Winter’s description is not congruent with a later cursory investigation of the pycnogonid mushroom bodies [91] and is also not in line with the original view held by Hanström [51], who tentatively assigned the term “corpora pedunculata” to the DPL that underlies the AB posteriorly (Table 1). It was beyond the scope of this study to identify a potential mushroom body homolog in the pycnogonid brain. Nonetheless, even at this stage, the direct targeting of the AMN by LO axon terminals does not support the interpretation of Winter [52], as it characterizes the AMN as first-order sensory neuropil instead of a higher-order integration center.

### Comparison to euchelicerate taxa

The present study contradicts the previously claimed presence of two parallel first-order VNs in pycnogonids [38, 53]. Instead, the pycnogonid visual pathway displays a simple serial layout, in which the R-cell axons project exclusively to a single VN, from where information is relayed by visual interneurons to the AB.

Fortuitously, a suite of recent investigations has systematically teased apart the array and connectivity of the VNs in almost all major arachnid taxa with modern techniques [27–29, 56, 79, 80], complementing a more comprehensive bulk of studies on spiders [59, 61, 92–94] and the marine horseshoe crabs [95–98]. Comparison with these works shows that the general layout of the visual system in pycnogonids is similar to the median (or principal) eyes of arachnids as well as the median eyes and the fused rudimentary median eyes of horseshoe crabs. Accordingly, also after revision of the results of Lehmann and colleagues [38], the visual pathway supports the widely assumed homology of pycnogonid eyes and euchelicerate median eyes [38, 99, 100].

However, one difference between pycnogonids and virtually all euchelicerate taxa is the presence of just one *versus* two serially arrayed VNs upstream of the (putative) connection to the AB (for recent overviews see, e.g., [29, 56]). Notably, an exception to this may be the rudimentary median eyes in horseshoe crabs, from which photoreceptive cells extend axonal projections via the first-order VN directly into the vicinity and into the AB [95–98]. Unfortunately, higher-order interneuron connections as shown here for the pycnogonid VN and AB are hitherto unstudied in horseshoe crabs. Beyond that, xiphosurans are also the only extant euchelicerate group that shares with pycnogonids the presence of two pairs of median eyes that project to the same VN, even though the rudimentary median eyes are externally hidden under the cuticle in adult horseshoe crabs [98, 99]. By contrast, other euchelicerate taxa possess only one pair (which may be reduced as well). During xiphosuran development, the rudimentary median eyes develop prior to hatching, whereas the median eyes become recognizable and functional only in the trilobite larval stage [101]. Remarkably, this sequential development mirrors the processes in pycnogonids, where the hatching protonymphon larva possesses only one pair of eyes and the second pair differentiates later during the post-larval phase [34, 102–104]. Accordingly, pycnogonid and xiphosuran median eyes share several features (number, aspects of their visual pathway, and developmental sequence) that are not found in other extant chelicerates and may therefore represent plesiomorphic features of the chelicerate ground pattern.

### Considerations in the context of chelicerate phylogeny

In spite of considerable progress over the last decades, several nodes in the phylogenetic tree of chelicerates are still under debate [1, 2]. Currently, one of the most contentious issues is the position of the marine Xiphosura. While some recent phylogenomic studies support their traditional placement as sister group of monophyletic terrestrial Arachnida [7–9], others recover them nested within the arachnid taxa [6, 10–12], thereby indicating multiple marine-terrestrial transitions within Chelicerata or a reconquering of marine habitats by xiphosurans. Putative morphological support for xiphosurans nested within arachnids has been—among others—derived from their adult visual system, which shares similarities with several arachnopulmonate taxa (scorpions, whip spiders, whip scorpions) [27–29]. Remarkably, these groups share interconnections between the VNs of the median and lateral eye pathways via specific R-cell projections, which are not reported for any other chelicerate group (see [29, 56]). However, although these similarities are doubtlessly striking, they may still reflect the plesiomorphic state of chelicerates that possess fully functional median *and* lateral eyes, as opposed to taxa with reduced median eyes (such as pseudoscorpions [80];) or (largely) reduced lateral eyes (as seen in opiliones or solifuges [56, 79];). The various eye losses in different chelicerate taxa pose considerable obstacles for phylogenetic and evolutionary interpretations based on the adult visual pathway, as the complexity of the latter is tightly linked to the types of eyes that are present. Once one of the eye types is largely reduced or lost, its affiliated VNs are likewise reduced or completely missing [56, 79, 80]. A similar correlation of eye loss and the absence of dedicated VNs is also found in blind representatives of mandibulate taxa [105–108]. Notably, this phenomenon can potentially even be seen within the Arachnopulmonata. A recent phylogenomic study leveraging first genomic data convincingly supports pseudoscorpions as sister group of scorpions [109]. But in line with the lack of median eyes, the pseudoscorpion visual system features no median VNs [80]. This logically precludes their interconnection via median eye R-cell projections to the lateral VNs and results in a visual pathway lacking the complex layout considered characteristic for arachnopulmonates.

In other words, characters of the chelicerate visual pathway that are functionally linked to the presence of both eye types need to be treated as non-independent from the latter. As such, however, they are phylogenetically uninformative in relation to taxa lacking one of the eye types. This highlights the need for additional eye-type-specific data classes that can be evaluated in a phylogenetic context (see section “Future perspectives: a neurodevelopmental approach to chelicerate visual system evolution”).

### New fossils and their potential impact on scenarios of chelicerate visual system evolution

In the last decade, new arthropod fossils with exceptional preservation of internal nervous system structures have been described [110–115], affording a rare opportunity to catch a fragmentary glimpse at putative ancestral layouts of the arthropod, chelicerate, and mandibulate visual pathways. Intriguingly, in the Leanchoiliidae, a group of the Cambrian “great appendage” arthropods variably assigned to the arthropod or chelicerate stem lineage [111, 115–117], connections from the forward- and sideward-directed eye pairs to the anterior brain were discovered [111, 114, 115]. In one specimen, these have been reconstructed to collectively target one VN located outside of the central brain neuropil (*Alalcomenaeus* sp., [111]), displaying some resemblance to the pycnogonid layout. However, a more recent study challenges this view and interprets the two eye pairs of other leanchoiliids to project to two different areas of the brain without specifying any VNs [115], concomitant with a reinterpretation of the pattern in *Alalcomenaeus* (Fig. 4 in [115]). This ready reinterpretation of *Alalcomenaeus* exemplarily illustrates that the identification of internal brain centers in fossils is challenging and to some extent ambiguous, in spite of exceptional neural tissue preservation. Objectively, the lack of resolution at the tissue level impedes unequivocal characterization of neuroanatomical elements and their interconnections within the brain, restricting reliable conclusions to gross features of brain anatomy. Somewhat disconcertingly, however, ambiguity even extends to the basic structural type of the external leanchoiliid eyes, which have been reported to be compound (to the inclusion of the forward-facing median eye pair) [111], single-lensed [115], or of unspecified type (e.g., [114]). Accordingly, notwithstanding the unusually good preservation of these new fossils, their impact on the reconstruction of the ancestral suite of chelicerate visual pathway elements remains speculative.

### Future perspectives: a neurodevelopmental approach to chelicerate visual system evolution

In the light of the topological congruence and corresponding developmental sequence in the two median eye pairs of xiphosurans and the basally branching pycnogonids, a promising future research avenue would be the study of anterior head patterning genes and the retinal determination gene network known to govern arthropod eye differentiation. To date, such studies are still rare in chelicerates and almost exclusively confined to spiders (e.g., [118, 119]; but see [120]), revealing differential gene expression patterns during the development of their different eye types [121, 122]. Accordingly, equivalent studies on the development of the two median eye pairs in pycnogonids and xiphosurans *versus* the single median eye pair of terrestrial euchelicerate taxa have the potential to uncover further eye type-specific similarities/discrepancies between the lineages and thus yield novel developmental data impacting current views on the evolution of the visual system in Chelicerata.

## Conclusions

Ground pattern reconstruction based on all extant families reveals remarkable neuroanatomical stasis in the pycnogonid visual pathway, which is consistently composed of two pairs of median eyes that target one VN from which information is relayed to the AB by catecholaminergic visual interneurons. This layout exhibits similarities to the median eye pathway in euchelicerates, in particular in xiphosurans, with which pycnogonids share the two median eye pairs that differentiate consecutively during development and connect to one VN upstream of the AB. Given multiple losses of median and/or lateral eyes in chelicerates, and the tightly linked reduction of visual processing centers, corresponding interconnections between median and lateral VNs in xiphosurans and arachnopulmonates need to be critically evaluated with regard to their phylogenetic information content, representing a plausible plesiomorphic condition of those taxa that have retained both eye types. Comparative studies on anterior head patterning genes and the retinal differentiation gene network governing eye development and the formation of anterior brain centers (such as median VNs and the AB), are a promising field for future research seeking to establish additional eye-type-specific commonalities and differences between the various chelicerate taxa. Notably, the inferred basal diversification of the pycnogonid crown group [73, 74] in the Ordovician or even earlier in conjunction with widely accepted stem group representatives (e.g., [123]) indicate that the pycnogonid lineage has undergone one of the earliest losses of the lateral eye pathway during arthropod evolution.

## Methods

### Specimen collection

Specimens were collected over several years in various locations, including marine research stations, such as Station Biologique de Roscoff (Bretagne, France) and Rothera Research Station (British Antarctic Survey, Antarctica) and cruises of research vessels, such as RV Polarstern [124]. An overview of all species investigated, specimen collection sites, and number of specimens studied with each method is provided in Additional file 1: Table S1.

### Micro-computed X-ray tomography

Specimens intended for internal anatomical study were fixed and stored in Bouin’s fluid (10% formaldehyde, 5% glacial acetic acid in saturated aqueous picric acid). Some animals that had been fixed and stored for several years either in borax-buffered 10% formaline in sea water or in 96% ethanol were exclusively used for external morphological investigation. Specimens were rinsed in phosphate-buffered saline (PBS; 1.86 mM NaH_2_PO_4_, 8.41 mM Na_2_HPO_4_, 175 mM NaCl, pH 7.4), transferred into deionized water, dehydrated via an ascending ethanol series, and incubated in a solution of 2% iodine (resublimated; Carl Roth; #X864.1) in 99.5% ethanol for 48–72 h at ambient temperature. After rinsing in 99.5% ethanol (3–4 × 10 min), specimens were either directly scanned in ethanol or critical point-dried using a Leica EM CPD300. Dried specimens were placed in plastic tubes for overview scans and subsequently attached to plastic welding rods with hot glue for detail scans. Scans were performed with an Xradia MicroXCT-200 (Carl Zeiss Microscopy) under 40 kV/200 μA/8 W or 30 kV/200 μA/6 W settings. Depending on specimen size and region of interest, a × 0.39, × 4, × 10, or × 20 objective was chosen. Exposure times were individually adjusted for each scan, ranging from 0.85 to 7 s. To reduce noise, binning 2 was applied during data acquisition. Tomography projections were reconstructed with the XMReconstructor software (Carl Zeiss Microscopy) with binning 1 (= full resolution) and TIFF format image stacks as output.

### Paraffin histology

Specimens of *Endeis spinosa* were fixed and stored in Bouin’s fluid until further use. Samples were rinsed in several changes of PBS, dehydrated in a graded ethanol series over 24 h, incubated overnight in a 1:1 mixture of ethanol and tetrahydrofuran (Carl Roth, #CP82.1), followed by pure tetrahydrofuran for 24 h and a 1:1 solution of tetrahydrofuran and paraffin (Carl Roth, #6643.1) for 24 h at 60 °C. Samples were then incubated for minimally 24 h in 100% paraffin at 60 °C and embedded in fresh paraffin. Sectioning was performed with a motorized Microm HM 360 rotary microtome at 8-μm thickness. Sections were Azan-stained according to Geidies [125] and subsequently mounted in Roti Histokitt II (Carl Roth, #T160.1).

### Specimen fixation, dissection, and vibratome sectioning for immunohistochemistry

Most specimens used for immunohistochemical studies were fixed overnight at 4 °C in 4% paraformaldehyde in sea water (PFA/SW; 16% methanol-free formaldehyde [Electron Microscopy Sciences, #15710] diluted 1:4 in 0.2 μm pore-filtered natural sea water), rinsed in PBS and either directly processed or transferred to cryoprotectant buffer (0.5× PBS, 300 g/L sucrose, 30 % (v/v) ethylene glycol, 10 g/L polyvinylpyrrolidone) for long-term storage at − 20 °C. Deviating from this standard procedure, samples intended for TH immunolabeling were fixed for 1 h only at room temperature (RT), as preliminary experiments confirmed previous reports of reduced or even complete loss of signal after longer fixation times (e.g., [26]). For histamine immunolabeling, specimens were pre-fixed for 4 h at RT in 4% EDAC (N-[3-dimethylaminopropyl]-N’-ethylcarbodiimide hydrochloride; Sigma-Aldrich; #03450) dissolved in filtered sea water or PBS, followed by post-fixation in PFA/PBS either for 4 h at RT or overnight at 4 °C. Alternatively, some specimens were pre-fixed in 4% EDAC/PBS overnight at 4 °C, followed by PFA/PBS post-fixation for 4 h at RT. These different fixation protocols have previously yielded positive results in various arthropod taxa, including chelicerates [61, 65, 81, 126].

For the study of brain whole mounts, the complete central nervous system was manually dissected in PBS under a stereomicroscope. For vibratome sectioning, specimens were coated in an aqueous 0.1% poly-L-lysine solution (Sigma-Aldrich, #P8920) and embedded in 8–10% low-gelling agarose (Sigma-Aldrich, #A9414) dissolved in deionized water. Sectioning was performed with a Zeiss Hydrax V50 vibratome; section thickness ranged from 50 to 100 μm, depending on species and specimen size.

### Immunohistochemistry

As standard immunohistochemical procedure, dissected brains and vibratome sections were rinsed in PBS at RT and permeabilized for ≥ 2 h in several changes of PBTx (PBS + 0.5% Triton-X + 1.5% dimethyl sulfoxide). Prior to incubation in primary and secondary antibodies/-sera, samples were blocked in PBTx + 5% normal goat serum (Thermo Fisher Scientific, #31873) for at least 1 h at RT. All primary and secondary antibodies/-sera were diluted in PBTx; incubation times lasted 72–120 h and were followed by rinsing in PBTx with gentle rotation for at least 6 h at RT and occasional extension overnight at 4 °C. Omission of primary antibodies/-sera in the procedure resulted in complete loss of signal.

In addition to this standard procedure, whole-mount brains of species with well-developed neural sheath (e.g., *Pycnogonum litorale*) were optionally exposed to Proteinase K (1× solution, Thermo Fisher Scientific, #C10617) in PBS for 15 min, rinsed in several changes of PBS and post-fixed in 4% PFA/PBS for 15 min at RT.

To improve the signal-to-noise ratio in histamine immunolabeling experiments, brains and vibratome sections were pre-incubated in unconjugated Fab fragments (donkey anti-rabbit; Table 2) in PBTx for 2–2.5 h at RT, rinsed in several quick changes of PBTx, post-fixed in 4% PFA/PBS for 15 min at RT, followed by the standard procedure.

**Table 2:** Antibodies/antisera, research resource identifiers and dilutions

Antibody/-serum	#RRID	Dilution
**Primary**		
Rabbit anti-5-hydroxytryptamin (serotonin), polyclonal, ImmunoStar, #20080	AB_572263	1:1000
Rabbit anti-histamine, polyclonal, Progen, #16043	AB_2892841	1:1000
Rabbit anti-Asn^13^-orcokinin, polyclonal, gift from Heinrich Dircksen (Stockholm University)	AB_2315017	1:1500
Rabbit anti-A-type Dip allatostatin I, polyclonal, Jena Bioscience, #ABD-062	AB_2314318	1:2000
Rabbit anti-proctolin, polyclonal, Jena Bioscience, #ABD-032	AB_2892840	1:1000
Mouse anti-tyrosine hydroxylase, IgG1 isotype, clone LNC1, ImmunoStar, #22941	AB_572268	1:300
Mouse anti-SYNORF1 (*Drosophila* synapsin-1), monoclonal, supernatant, Developmental Studies Hybridoma Bank, #3C11	AB_528479	1:100
Mouse anti-acetylated tubulin IgG 2b isotype, clone 6-11 B-1, Sigma-Aldrich, #T6793	AB_477585	1:200–300
Rat anti-alpha-tubulin IgG2a isotype, clone YL1/2, Thermo Fisher Scientific, #MA1-80017	AB_2210201	1:500
**Secondary**		
Alexa Fluor488 goat anti-mouse IgG (H + L), polyclonal, Thermo Fisher Scientific, #A11001	AB_2534069	1:250
Cy3-AffiniPure goat anti-mouse IgG (H + L), polyclonal, Jackson ImmunoResearch Labs, #115-165-166	AB_2338692	1:250
Alexa Fluor647-AffiniPure goat anti-mouse IgG (H + L), polyclonal, Jackson ImmunoResearch Labs, #115-605-166	AB_2338914	1:250
Alexa Fluor488-AffiniPure goat anti-rabbit IgG (H + L), polyclonal, Jackson ImmunoResearch Labs, #111-545-144	AB_2338052	1:250
Alexa Fluor594 goat anti-rabbit IgG (H + L), polyclonal, Thermo Fisher Scientific, #A11012	AB_2534079	1:250
Alexa Fluor647-AffiniPure goat anti-rat IgG (H + L), polyclonal, Jackson ImmunoResearch Labs, #112-605-167	AB_2338404	1:250
unconjugated AffiniPure Fab fragment donkey anti-rabbit IgG (H + L), polyclonal, Jackson ImmunoResearch Labs, #711-007-003	AB_2340587	1:25

### Specification of primary antibodies/-sera

For full details of primary and secondary antibodies/-sera and their dilutions, see Table 2.

The monoclonal mouse anti-SYNORF1 antibody (deposited at the Developmental Studies Hybridoma Bank by E. Buchner, University Hospital Würzburg) was raised against a *Drosophila melanogaster* GST-synapsin fusion protein and has proved a useful marker of synaptic neuropil across many arthropod taxa, including various chelicerates [81, 127, 128].

A monoclonal mouse antibody against acetylated alpha-tubulin was used to visualize cytoskeletal microtubules. It has been previously applied to successfully trace nerves and tracts in the pycnogonid ventral nerve cord [32, 129, 130]. For double labeling of tubulin-rich structures and synaptic neuropil regions, a monoclonal rat antibody against alpha-tubulin was used, recognizing the tyrosinated form of the alpha subunit of tubulin in a wide range of metazoan taxa according to the manufacturer’s specifications.

The biogenic amine histamine (HIS) acts as inhibitory neurotransmitter in arthropod photoreceptive cells [63, 64]. The polyclonal rabbit antiserum was raised against HSA-conjugated histamine and has been previously applied in chelicerate taxa with success [65, 93].

The biogenic amine 5-hydroxytryptamin (5HT, serotonin) is a widespread neurotransmitter in metazoans and was labeled with a polyclonal rabbit antiserum raised against serotonin coupled to BSA with PFA. The antiserum has been previously applied in various arthropod taxa, including pycnogonids [26, 129, 131, 132].

Tyrosine hydroxylase (TH) is the rate-limiting enzyme in the synthesis of catecholamines (dopamine, adrenaline, noradrenaline) [69], of which dopamine represents an important neurotransmitter in the arthropod nervous system. A monoclonal mouse antibody raised against purified TH from rat PC12 cells was used, having been prior to this study successfully applied in spiders [70].

Orcokinins (ORCO) are a conserved neuropeptide family that is expressed in the nervous system of many arthropods, including chelicerates (e.g., [133–135]). The polyclonal rabbit Asn^13^-orcokinin antiserum used in this study was kindly provided by H. Dircksen (Stockholm University). It was raised against Asn^13^-orcokinin coupled by glutaraldehyde to bovine thyroglobulin [136].

The neuropeptide proctolin (PROC) is expressed in neurons of the central nervous system in different arthropod groups, including chelicerates [60, 137, 138]. In this study, it was labeled with a polyclonal rabbit antiserum raised against proctolin coupled to glutaraldehyde/polylysine.

The polyclonal rabbit antiserum against allatostatin (AST) used in this study was raised against A-type *Diploptera punctata*-allatostatin I (APSGAQRLYGFGL amide) coupled to bovine thyroglobulin with glutaraldehyde [139]. Allatostatins form a large neuropeptide family and have been shown to be expressed in numerous neurons and neuropil regions of various arthropod groups, including the AB in spiders [60, 126, 139, 140].

### Fluorescent dye backfills

The lipophilic fluorescent dye DiI (1,1’-Dioctadecyl-3,3,3’,3’-tetramethyl-indocarboncyanin-perchlorate, Sigma-Aldrich, #42364) was used to trace afferents from the eyes’ R-cells and the sensory cells in the LO of *Endeis spinosa*. PFA-fixed samples were used, having been previously shown to be suitable for lipophilic dye tracing in arthropod brains [27, 79, 141, 142]. Specimens were placed in PBS and after manual perforation of an eye lens or the center of the LO with a sharpened tungsten tip, a small flake of DiI was externally applied to the damaged area. Approximately 1 h after dye application, the successful begin of the backfill was briefly checked under epifluorescence. Specimens were left overnight at 4 °C or alternatively at RT, fixed in PFA/PBS for 30 min, and rinsed in PBS. The brain was dissected with the ocular tubercle still attaching and post-fixed for another 30 min in PFA/PBS.

### Nuclear staining and mounting of samples

The nucleic acid marker Hoechst 33342 (Thermo Fisher Scientific, #H1399, 1 μg/mL in PBS) was applied after all other labeling procedures. Incubation lasted minimally 1 h at RT and was occasionally extended overnight at 4 °C. After rinsing in PBS, samples were transferred into non-hardening Vectashield® Mounting Medium (Vector Laboratories, Inc. #H-1000, RRID:AB_2336789) and placed on microscopic slides. To avoid squeezing of whole-mount brains, small pieces of Surgident periphery wax were placed under the cover slip corners and gradually compressed under a stereomicroscope. The flexible spacers enabled fine adjustment of the orientation of the whole-mount brains via horizontal movement of the cover slip.

### Data documentation and analysis:

Histological sections were documented with a Nikon Eclipse 90i epifluorescence microscope, equipped with a Nikon DS Fi3 camera. XY-tiling and combination into images with extended depth of field was managed in the accompanying NIS Elements AR software (ver. 5.02, Nikon Corporation, Tokyo, Japan, RRID:SCR_014329).

Confocal laser scanning microscopy (CLSM) was performed with a Leica DMI 6000 CS microscope coupled to a Leica TCS SP5 II scan unit (RRID:SCR_018714). Laser lines were chosen according to the excitation spectra of the fluorochromes applied (405 nm → Hoechst; 488 nm → Alexa Fluor 488; 543 nm → Cy3; 594 nm → Alexa Fluor 594; 633 nm → Alexa Fluor 647). The Z-increment between optical planes ranged between 0.50 and 1.50 μm, depending on objective used and required resolution.

Analysis and visualization of the CLSM and μCT image stacks was performed with the software packages Imaris (ver. 7.00; Bitplane AG, Zurich, Switzerland, RRID:SCR_007370) and Amira (ver. 5.6; FEI Visualization Sciences Group, RRID:SCR_007353). Software tools were applied as previously described (Imaris: [143, 144]; Amira: [145]), including (i) the use of optical clipping planes to remove non-target structures, (ii) the generation of 3D-curved optical sections by combination of several oblique slicers, and (iii) the manual segmentation of structures of interest for improved depiction by selective coloration. Movies were generated in Imaris.

### Data presentation and terminology

Global contrast, brightness, and sharpness of images were adjusted using Adobe Photoshop (ver. 12.1, Adobe Systems Incorporated, San Jose, CA, USA, RRID:SCR_014199). Figures were assembled with Adobe Illustrator (ver. 15.1, Adobe Systems Incorporated, RRID:SCR_010279).

Neuroanatomical terminology follows for the most part Richter and colleagues [15]. Species names are updated according to PycnoBase [146]. A list of abbreviations used in this study to designate neuroanatomical structures is provided in Table 3.

**Table 3:** List of abbreviations used in the study

5HT	5-hydroxytryptamine (serotonin)	NUC	Nuclear staining
AB	Arcuate body	ONV	Optic nerve
ABL	Lower arcuate body layer	ORCO	Orcokinin
ABU	Upper arcuate body layer	PBS	Phosphate-buffered saline
AE	Anterior eye	PBTx	PBS + Triton X-100
AEL	Anterior eye lens	PE	Posterior eye
AF	Autofluorescence	PEL	Posterior eye lens
AMN	Antero-median neuropil	PFA	Paraformaldehyde
AMT	Antero-median tract	PNV	Proboscis nerve
AST	Allatostatin	PROC	Proctolin
BR	Brain	R-cell	Retinula cell
BRN	Central brain neuropil	RT	Room temperature
CENV	(dorsal) cephalon nerve	RTA	R-cell axon (bundle)
CHNV	Celiphore nerve	SC	Soma cortex
CLSM	Confocal laser scan microscopy	SVD	Sub-visual domain
PDL	Dorso-posterior lobe	SW	Sea water
HIS	Histamine	SYN	Synapsin
ir	Immunoreactive	TH	Tyrosine hydroxylase
LO	Lateral sense organ	TUB	Tubulin
LONV	Lateral sense organ nerve	VN	Visual neuropil
LT	Lateral thickening	μCT	Micro-computed X-ray tomography
MIP	Maximum intensity projection		

## Supplementary Information

**Additional file 1: Table S1**: List of species studied, collection sites and methods applied.

**Additional file 2: Figure S1**: Selected details of the sense organs and protocerebral structures in *E. spinosa*. Extended optical sections of immunolabeled samples (MIP). **A:** Tubulin (TUB) labeling, para-sagittal section through the ocular tubercle. A subepidermal network of neurite bundles (arrows point to selected examples) spreads in the ocular tubercle and dorsal cephalon, being connected to the lateral thickening via a postero-lateral nerve (black arrowhead). R-cell axon bundles (white arrowheads) and the optic nerve (star) have been severed during vibratome sectioning. The asterisks mark muscle attachment sites. **B:** Tyrosine hydroxylase (TH, magenta) and histamine (HIS, green), para-sagittal section through anterior portion of the brain. TH-ir somata of type 1 interneurons (arrowheads) are found adjacent to the visual neuropil. Note indications for TH-ir neurites in the optic nerve (arrows). **C**,**D:** Orcokinin (ORCO), mid-sagittal and oblique horizontal brain sections (**C** and **D**, respectively). **C:** Orcokinin-ir neurons (arrowheads) send projections through the antero-median tract (arrows) to the lower layer of the arcuate body. Note orcokinin labeling of the antero-median neuropil. **D:** “Naked” orcokinin-ir axonal projections run through the antero-median tract and form dense synaptic varicosities upon entry (arrow) into the lower arcuate body layer. Note scattered synaptic varicosities (arrowhead) also in the upper arcuate body layer. **E:** Serotonin (5HT), oblique horizontal brain section. Serotonin-ir axons from the antero-median tract project into the lower arcuate body layer (arrow) and form dense synaptic varicosities. Note also a loose network of synaptic varicosities in the dorso-posterior lobe. Abbreviations: AB – arcuate body; ABL – lower arcuate body layer; ABU – upper arcuate body layer; AE – anterior eye; AMN – antero-median neuropil; AMT – antero-median tract; DPL – dorso-posterior lobe; LO – lateral sense organ; PE – posterior eye; RTA – R-cell axons; SVD – sub-visual domain, VN – visual neuropil.

**Additional file 3: Movie S1**: Serotonin and tyrosine hydroxylase expression in the anterior protocerebral region of *Endeis spinosa*. Tyrosine hydroxylase (magenta) and serotonin (green) immunolabeling with nuclear counterstain (gray), 3D volume of anterior protocerebral region (blend mode). The brain is shown in anterior view (in terms of neuraxis) and rotates counterclockwise to show the spatial arrangements of the visual neuropil, arcuate body (upper and lower layer), dorso-posterior lobe and antero-median tract. Note that the arcuate body is antero-dorsally covered by the soma cortex (see start of the movie). Abbreviations used in the movie: ABL – lower arcuate body layer; ABU – upper arcuate body layer; AMT – antero-median tract; DPL – dorso-posterior lobe; VN – visual neuropil.

**Additional file 4: Movie S2**: Serotonin expression in the anterior protocerebral region of *Endeis spinosa*. Serotonin immunolabeling, 3D volume of anterior protocerebral region (blend mode). The brain is shown in anterior view (in terms of neuraxis) and rotates clockwise to show the spatial arrangement of the lower arcuate body layer, antero-median tract and the group of antero-dorsal neurons that project past the antero-median tract into the central brain neuropil. Note the ventral neurons with projections that loop into the antero-median tract and contribute to the dense synaptic varicosities in the lower arcuate body layer. Abbreviations used in the movie: AB – arcuate body; ABL – lower arcuate body layer; AMT – antero-median tract; DPL – dorso-posterior lobe.

**Additional file 5: Movie S3**: Tyrosine hydroxylase expression in the anterior protocerebral region of *Endeis spinosa*. Tyrosine hydroxylase immunolabeling, 3D volume of anterior protocerebral region (blend mode). The brain is shown in anterior view (in terms of neuraxis) and rotates clockwise to show the spatial arrangement of the TH-ir projections and synaptic varicosities in the visual neuropil and upper arcuate body. The somata of anterior type 1 and antero-ventral type 2 neurons are highlighted as the brain rotates. Note also medial projections from the sub-visual domain into the central brain neuropil. Abbreviations used in the movie: ABU – upper arcuate body layer; VN – visual neuropil.

**Additional file 6: Figure S2**: Tyrosine hydroxylase (TH) expression in the anterior protocerebrum of various pycnogonid families. Horizontal sections of immunolabeled samples (MIP). The left image column shows extended sections that include the visual neuropil, sub-visual domain and arcuate body. In the central image column, major parts of the apical visual neuropil have been removed by a clipping plane. The right image column depicts the apical visual neuropil only. White arrows mark selected type 2 neurons, white arrowheads indicate type 1 neurons. In some samples (**A**,**C**,**E**), some neurites in the optic nerve are visible (black arrowheads). Black arrows indicate weakly or strongly labeled projections from the sub-visual domain into the underlying central brain neuropil. Light blue arrowheads point to somata of dorsal neurons extending projections into more posterior brain regions (not shown), sometimes looping anteriorly around the narrow median portion of the arcuate body (**B**,**D**-**F**). **A:** *Pycnogonum litorale* (Pycnogonidae). Note weak TH signal in the visual neuropil and the presumptive type 1 neurons. Small white arrows highlight small TH-ir somata in the lateral thickening. **B:** *Anoplodactylus australis* (Phoxichilidiidae). **C:** *Endeis spinosa* (Endeidae). **D:** *Pallenopsis* sp. (Pallenopsidae). Note weak TH signal in the visual neuropil and the presumptive type 1 neurons. **E:** *Austropallene cornigera* (“Callipallendidae”). **F:** *Nymphon tenuipes* (Nymphonidae). Abbreviations: AB – arcuate body; SVD – sub-visual domain, VN – visual neuropil.

**Additional file 7: Figure S3**: Protocerebral sense organs and their brain connections in *Phoxichilidium femoratum* (Phoxichilidiidae). Extended optical sections (**A**,**E**) and 3D-curved optical sections (**B**-**D**) of immunolabeled samples (MIP). Yellow arrowheads point to the optic nerve branch extending to the antero-median neuropil. **A:** Tubulin (TUB, green) and synapsin (SYN, magenta) with nuclear counterstain and autofluorescence (AF + NUC, gray; left image only), para-sagittal section. The tubulin-labeled lateral sense organ and its nerve (arrow) to the lateral thickening as well as the proximal portion of the nerve (black arrowhead) connecting to the subepidermal neurite network of the ocular tubercle have been segmented and highlighted in different colors (red and yellow, respectively). **B**,**C:** Tubulin (green) and synapsin (magenta), detail of the optic nerve as it enters the brain, para-sagittal section. In the optic nerve, the neurites from the lateral sense organ and the axons targeting the visual neuropil remain separated as they enter the soma cortex. **D:** Tubulin (magenta; left image only) and orcokinin (ORCO, green) with nuclear counterstain and autofluorescence (gray), para-sagittal section. A subset of orcokinin-ir lateral sense organ cells (white arrows) appears to project orcokinin-ir projections (white arrowheads) through the lateral sense organ nerve toward the brain. Note absence of orcokinin labeling in the visual neuropil. **E:** Tubulin (magenta) and serotonin (5HT, green) with nuclear counterstain (gray, upper image only), cross section through anterior brain region. Note additional branch from the optic nerve that passes the antero-median neuropil (red arrowhead). The antero-median tract shows strong serotonin signal and also the antero-median neuropil is weakly labeled. Abbreviations: AE – anterior eye; AEL – anterior eye lens; AMN – antero-median neuropil; AMT – antero-median tract; BRN – brain neuropil; LO – lateral sense organ; LONV – lateral sense organ nerve; LT – lateral thickening; PE – posterior eye; PEL – posterior eye lens; RTA – R-cell axons; VN – visual neuropil.

**Additional file 8: Figure S4**: The eyes and lateral sense organ in all pycnogonid families. Volume renderings of the ocular tubercle, μCT scans in lateral view. If the eye lenses or lateral sense organs where not clearly discernible by external inspection alone, their presence was additionally confirmed by the study of the internal anatomy in the scans. **A:** *Austrodecus glaciale* (Austrodecidae). **B:** *Rhynchothorax australis* (Rhynchothoracidae). **C:** *Pycnogonum litorale* (Pycnogonidae). Note absence of the lateral sense organ. **D:** *Colossendeis angusta* (Colossendeidae). Note absence of the eyes. **E:** *Endeis spinosa* (Endeidae). **F:** *Phoxichilidium femoratum* (Phoxichilidiidae). **G:** *Pallenopsis* cf. *aulaeturcarum* (Pallenopsidae). **H:** *Achelia echinata* (Ammotheidae). **I:** *Ammothea longipes* (Ammotheidae). **J:** *Tanystylum orbiculare* (Ammotheidae). **K:** *Ascorhynchus ramipes* (Ascorhynchidae). Note upward shift of the posterior eye and the unusual position of the lateral sense organ below the latter. **L:** *Nymphon gracile* (Nymphonidae). **M:** *Callipallene tiberii* (“Callipallenidae”). **N:** *Pallenella* sp. (“Callipallenidae”). **O:** *Parapallene avida* (“Callipallenidae”). **P:** *Stylopallene cheilorhynchus* (“Callipallenidae”). Note external subdivision of the eye lenses. Abbreviations: AEL – anterior eye lens; LO – lateral sense organ; PEL – posterior eye lens.

**Additional file 9: Figure S5**: Histamine expression in eyes and first-order visual neuropil of Ammotheidae and Phoxichilidiidae. Extended optical sections of immunolabeled samples (MIP). **A**,**C**,**E:** Tubulin (TUB, magenta; left images only) and histamine (HIS, green) with nuclear counterstain and autofluorescence (AF + NUC, gray), horizontal sections through the ocular tubercle. Note absence of histamine labeling in the lateral sense organ. **B**,**D**,**F:** Histamine (green) with nuclear counterstain (gray, left images only), horizontal sections through the anterior protocerebral region. Note absence of histamine labeling in the median area housing the antero-median neuropil. **A**,**B:** *Achelia echinata* (Ammotheidae). **C**,**D:** *Tanystylum orbiculare* (Ammotheidae). **E**,**F:** *Phoxichilidium femoratum* (Phoxichilidiidae). Abbreviations: AE – anterior eye; LO – lateral sense organ; LONV – lateral sense organ nerve; LT – lateral thickening; PE – posterior eye; RTA – R-cell axons; VN – visual neuropil.

**Additional file 10: Figure S6**: The first-order visual neuropil in various pycnogonid families. Tubulin (TUB, green) and synapsin (SYN, magenta) immunolabeling with nuclear counterstain (NUC, gray; except **A** and **B**), optical cross sections through the anterior protocerebral region. **A:** *Austrodecus glaciale* (Austrodecidae). **B:** *Rhynchothorax australis* (Rhynchothoracidae). **C:** *Pycnogonum litorale* (Pycnogonidae). **D:** *Endeis spinosa* (Endeidae). **E:** *Phoxichilidium femoratum* (Phoxichilidiidae). **F:** *Pallenopsis* sp. (Pallenopsidae). **G:** *Achelia echinata* (Ammotheidae). **H:** *Callipallene brevirostris* (“Callipallenidae”). **I:** *Nymphon gracile* (Nymphonidae). Abbreviations: BRN – brain neuropil; VN – visual neuropil.

**Additional file 11: Figure S7**: Selected details of protocerebral structures in Ammotheidae and Nymphonidae. Optical sections of immunolabeled samples (MIP). **A:** *Achelia echinata*, tyrosine hydroxylase (TH, magenta) with nuclear counterstain (NUC, gray; upper image only), cross section through anterior protocerebral region. TH-ir somata of type 1 interneurons (arrowheads) are located next to the TH-ir visual neuropil. Note also TH labeling in the sub-visual domain underlying the visual neuropil. **B:** *Nymphon* cf. *multituberculatum*, TH (magenta; upper and middle images) and serotonin (5HT, green; middle and lower images), extended horizontal section through anterior protocerebral region. Note TH and serotonin co-labeling in the visual neuropil. **C:** *Nymphon gracile*, serotonin, horizontal section through the antero-median tract and arcuate body. Serotonin-ir axons of ventral neurons project through the antero-median tract, bifurcate upon entry into the lower arcuate body layer (arrows) and form dense synaptic varicosities in its lateral arms. Note fine (and in part columnar) collaterals extending into the upper arcuate body layer (arrowheads). Abbreviations: ABL – lower arcuate body layer; ABU – upper arcuate body layer; AMT – antero-median tract; BRN – brain neuropil; SVD – sub-visual domain; VN – visual neuropil.

**Additional file 12: Figure S8**: The antero-median neuropil and antero-median tract in various pycnogonid families. **A**-**C:** Tubulin (TUB, green) and synapsin (SYN, magenta) immunolabeling with nuclear counterstain (NUC, gray), optical cross sections through the anterior protocerebral region. Yellow arrowheads indicate the branch of the optic nerve looping toward the antero-median neuropil. **A:** *Achelia echinata* (Ammotheidae). **B:** *Pallenopsis* sp. (Pallenopsidae). **C:** *Nymphon gracile* (Nymphonidae). Note that the antero-median tract is not readily discernible in cross section, due to its rather diffuse, neuropil-rich nature in Nymphonidae (compare to Fig. 9E,F). **D**-**F:** Orcokinin (ORCO) immunolabeling, extended optical sections through the anterior protocerebral region (MIP). White arrows point to orcokinin-ir somata of anterior neurons that contribute neurites to the antero-median neuropil. Yellow arrowheads mark orcokinin-ir projections in the optic nerve branch extending to the antero-median neuropil. **D:** *Tanystylum orbiculare* (Ammotheidae). Note absence of distinct labeling in the antero-median tract. **E:** *Phoxichilidium femoratum* (Phoxichilidiidae). Note relatively weak labeling in the antero-median neuropil. Black arrowheads mark orcokinin-ir somata in the lateral thickening. **F:** *Callipallene brevirostris* (“Callipallenidae”). Note distinct orcokinin-ir projections (yellow arrowheads) from the optic nerve to the antero-median neuropil and strong signal in the antero-median neuropil as well as the antero-median tract. Abbreviations: AMN – antero-median neuropil; AMT – antero-median tract; BRN – brain neuropil; LT – lateral thickening.

**Additional file 13: Figure S9**: Serotonin expression in antero-median tract and arcuate body of various pycnogonid families. Serotonin (5HT) immunolabeling, extended horizontal sections through the brain (MIP). Arrowheads point to selected somata of ventral neurons that send projections along the antero-median tract into the arcuate body. **A:** *Anoplodactylus pygmaeus* (Phoxichilidiidae). **B:** *Phoxichilidium femoratum* (Phoxichilidiidae). **C:** *Pallenopsis* sp. (Pallenopsidae). Note relatively diffuse, neuropil-rich nature of the antero-median tract. **D:** *Tanystylum orbiculare* (Ammotheidae). **E:** *Achelia echinata* (Ammotheidae). **F:** *Ammothella biunguiculata* (Ammotheidae). Note relatively diffuse, neuropil-rich nature of the antero-median tract. **G:** *Ascorhynchus auchenicus* (Ascorhynchidae). **H:** *Callipallene brevirostris* (“Callipallenidae”). **I:** *Austrodecus glaciale* (Austrodecidae). Due to long-term PFA storage of the available material, the signal is extremely weak but still points to the presence of the serotonin-ir antero-median tract and lower arcuate body layer. Abbreviations: AB – arcuate body; AMT – antero-median tract; DPL – dorso-posterior lobe.

**Additional file 14: Table S2**: Overview of protocerebral sense organs and brain structures identified across pycnogonid families, with details on immunoreactivity for neuroactive substances.

## Data Availability

All data generated or analyzed during this study are included in this published article and its supplementary information files.
