# Supplementary figures and images for: The visual pathway in sea spiders (Pycnogonida) displays a simple serial layout with similarities to the median eye pathway in horseshoe crabs
Source: BMC Biol. 2022 Jan 28;20:27. doi: 10.1186/s12915-021-01212-z (PMC8796508; doi:10.1186/s12915-021-01212-z)

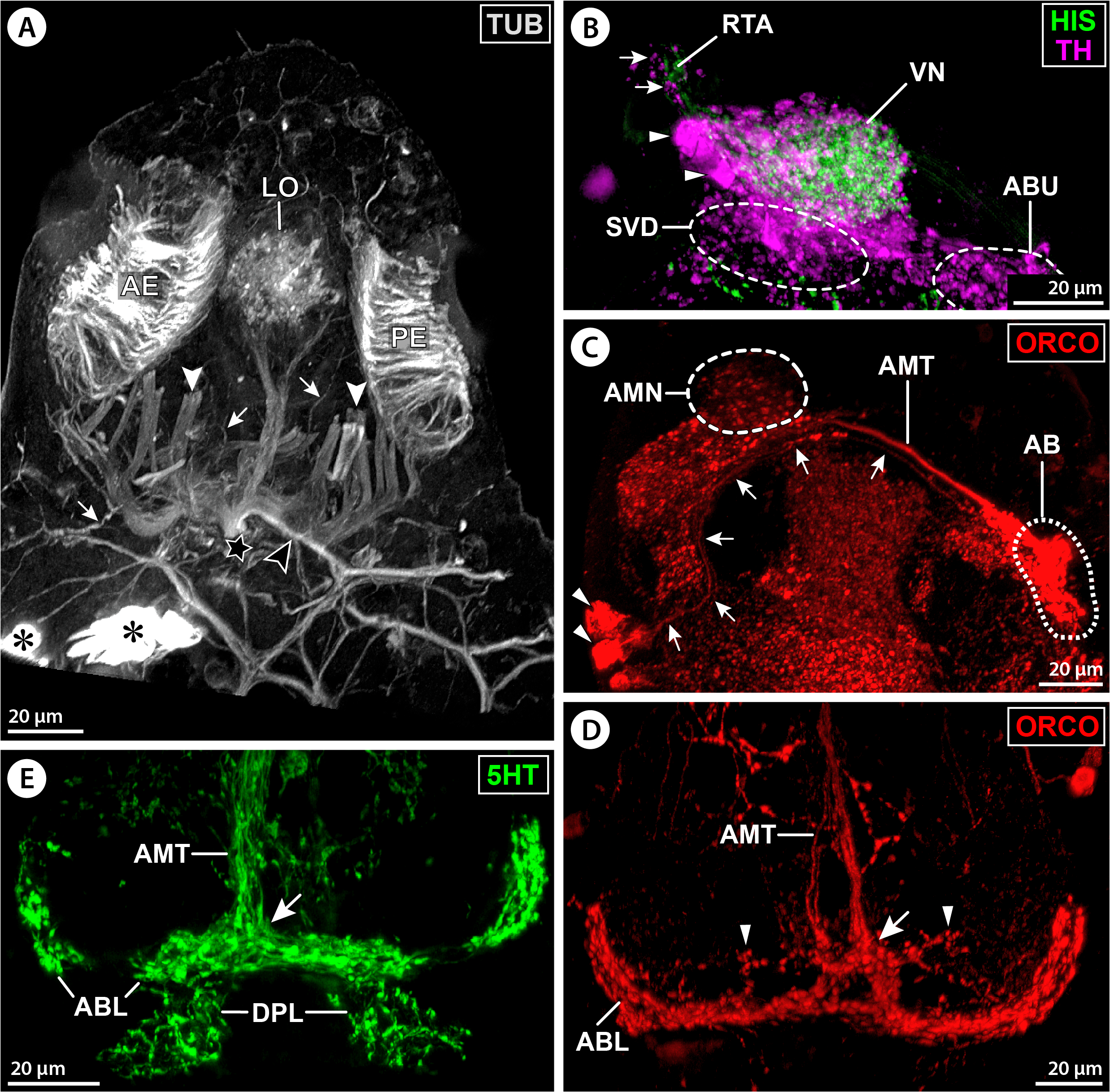

Supplement: Supplementary file 2 — Additional file 2: Figure S1: Selected details of the sense organs and protocerebral structures in E. spinosa. Extended optical sections of immunolabeled samples (MIP). A: Tubulin (TUB) labeling, para-sagittal section through the ocular tubercle. A subepidermal network of neurite bundles (arrows point to selected examples) spreads in the ocular tubercle and dorsal cephalon, being connected to the lateral thickening via a postero-lateral nerve (black arrowhead). R-cell axon bundles (white arrowheads) and the optic nerve (star) have been severed during vibratome sectioning. The asterisks mark muscle attachment sites. B: Tyrosine hydroxylase (TH, magenta) and histamine (HIS, green), para-sagittal section through anterior portion of the brain. TH-ir somata of type 1 interneurons (arrowheads) are found adjacent to the visual neuropil. Note indications for TH-ir neurites in the optic nerve (arrows). C,D: Orcokinin (ORCO), mid-sagittal and oblique horizontal brain sections (C and D, respectively). C: Orcokinin-ir neurons (arrowheads) send projections through the antero-median tract (arrows) to the lower layer of the arcuate body. Note orcokinin labeling of the antero-median neuropil. D: “Naked” orcokinin-ir axonal projections run through the antero-median tract and form dense synaptic varicosities upon entry (arrow) into the lower arcuate body layer. Note scattered synaptic varicosities (arrowhead) also in the upper arcuate body layer. E: Serotonin (5HT), oblique horizontal brain section. Serotonin-ir axons from the antero-median tract project into the lower arcuate body layer (arrow) and form dense synaptic varicosities. Note also a loose network of synaptic varicosities in the dorso-posterior lobe. Abbreviations: AB – arcuate body; ABL – lower arcuate body layer; ABU – upper arcuate body layer; AE – anterior eye; AMN – antero-median neuropil; AMT – antero-median tract; DPL – dorso-posterior lobe; LO – lateral sense organ; PE – posterior eye; RTA – R-cell axons; SVD – sub [file 12915_2021_1212_MOESM2_ESM.tif]

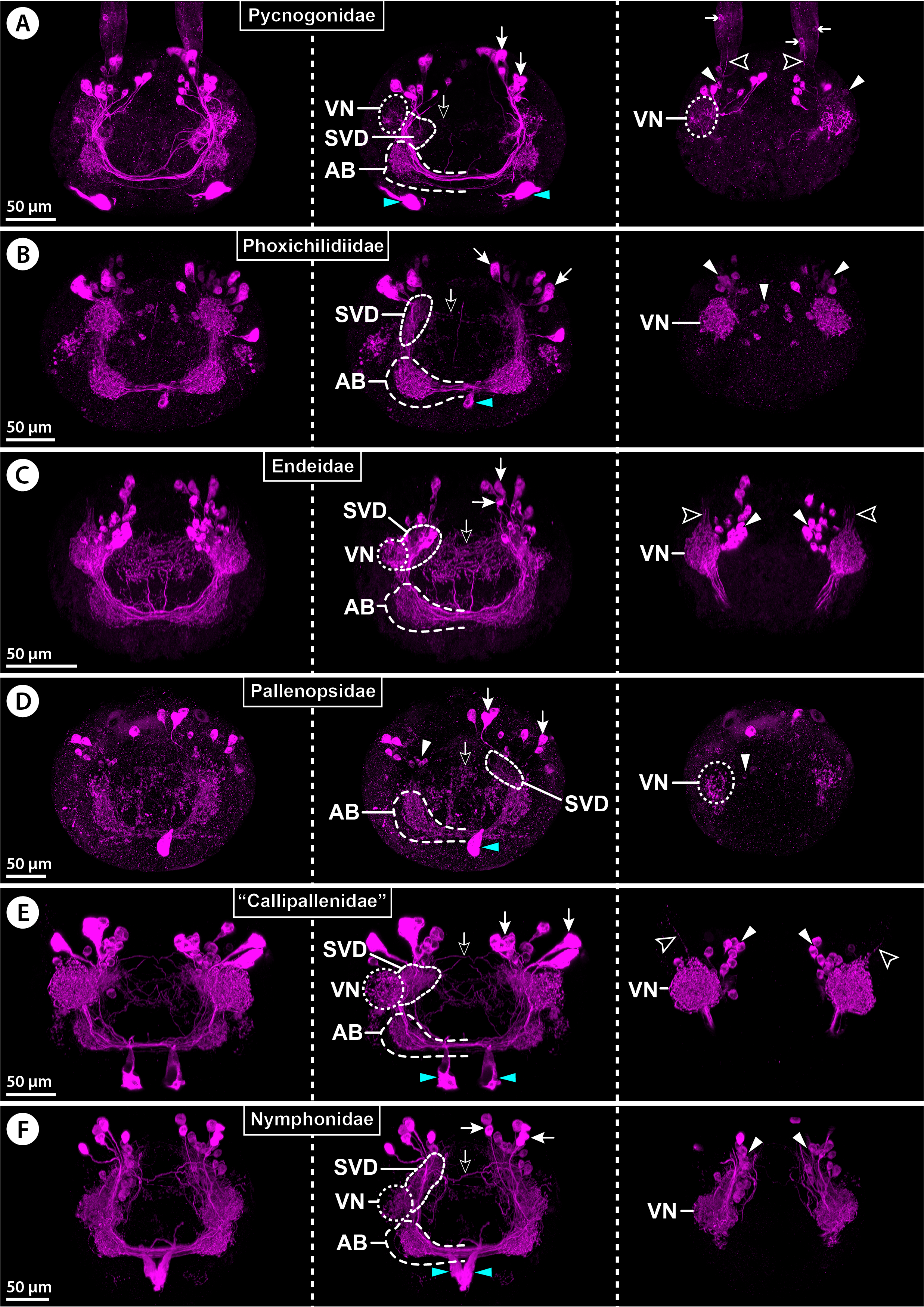

Supplement: Supplementary file 6 — Additional file 6: Figure S2: Tyrosine hydroxylase (TH) expression in the anterior protocerebrum of various pycnogonid families. Horizontal sections of immunolabeled samples (MIP). The left image column shows extended sections that include the visual neuropil, sub-visual domain and arcuate body. In the central image column, major parts of the apical visual neuropil have been removed by a clipping plane. The right image column depicts the apical visual neuropil only. White arrows mark selected type 2 neurons, white arrowheads indicate type 1 neurons. In some samples (A,C,E), some neurites in the optic nerve are visible (black arrowheads). Black arrows indicate weakly or strongly labeled projections from the sub-visual domain into the underlying central brain neuropil. Light blue arrowheads point to somata of dorsal neurons extending projections into more posterior brain regions (not shown), sometimes looping anteriorly around the narrow median portion of the arcuate body (B,D-F). A: Pycnogonum litorale (Pycnogonidae). Note weak TH signal in the visual neuropil and the presumptive type 1 neurons. Small white arrows highlight small TH-ir somata in the lateral thickening. B: Anoplodactylus australis (Phoxichilidiidae). C: Endeis spinosa (Endeidae). D: Pallenopsis sp. (Pallenopsidae). Note weak TH signal in the visual neuropil and the presumptive type 1 neurons. E: Austropallene cornigera (“Callipallendidae”). F: Nymphon tenuipes (Nymphonidae). Abbreviations: AB – arcuate body; SVD – sub-visual domain, VN – visual neuropil. [file 12915_2021_1212_MOESM6_ESM.tif]

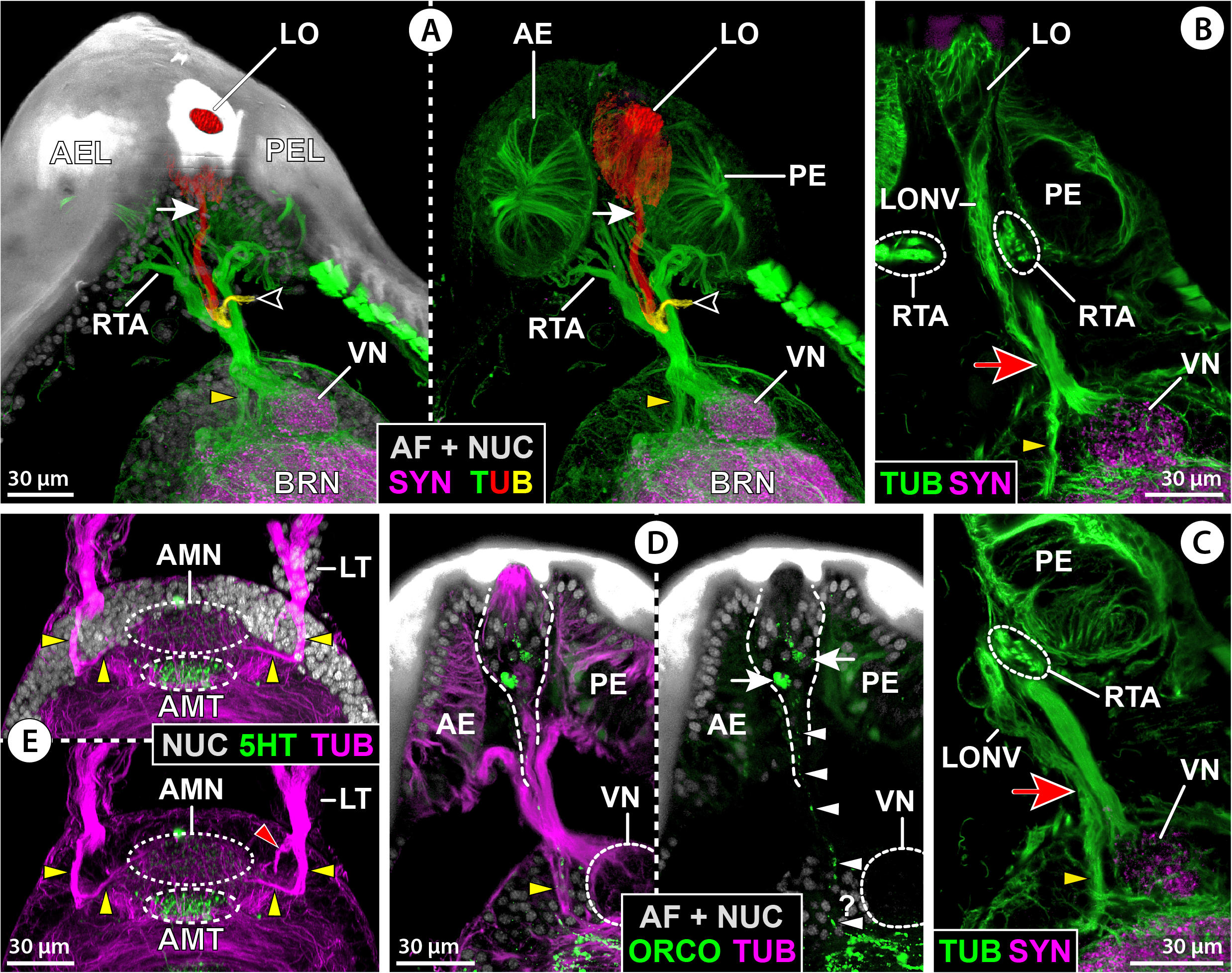

Supplement: Supplementary file 7 — Additional file 7: Figure S3: Protocerebral sense organs and their brain connections in Phoxichilidium femoratum (Phoxichilidiidae). Extended optical sections (A,E) and 3D-curved optical sections (B-D) of immunolabeled samples (MIP). Yellow arrowheads point to the optic nerve branch extending to the antero-median neuropil. A: Tubulin (TUB, green) and synapsin (SYN, magenta) with nuclear counterstain and autofluorescence (AF + NUC, gray; left image only), para-sagittal section. The tubulin-labeled lateral sense organ and its nerve (arrow) to the lateral thickening as well as the proximal portion of the nerve (black arrowhead) connecting to the subepidermal neurite network of the ocular tubercle have been segmented and highlighted in different colors (red and yellow, respectively). B,C: Tubulin (green) and synapsin (magenta), detail of the optic nerve as it enters the brain, para-sagittal section. In the optic nerve, the neurites from the lateral sense organ and the axons targeting the visual neuropil remain separated as they enter the soma cortex. D: Tubulin (magenta; left image only) and orcokinin (ORCO, green) with nuclear counterstain and autofluorescence (gray), para-sagittal section. A subset of orcokinin-ir lateral sense organ cells (white arrows) appears to project orcokinin-ir projections (white arrowheads) through the lateral sense organ nerve toward the brain. Note absence of orcokinin labeling in the visual neuropil. E: Tubulin (magenta) and serotonin (5HT, green) with nuclear counterstain (gray, upper image only), cross section through anterior brain region. Note additional branch from the optic nerve that passes the antero-median neuropil (red arrowhead). The antero-median tract shows strong serotonin signal and also the antero-median neuropil is weakly labeled. Abbreviations: AE – anterior eye; AEL – anterior eye lens; AMN – antero-median neuropil; AMT – antero-median tract; BRN – brain neuropil; LO – lateral sense organ; LONV – lateral sense organ nerv [file 12915_2021_1212_MOESM7_ESM.tif]

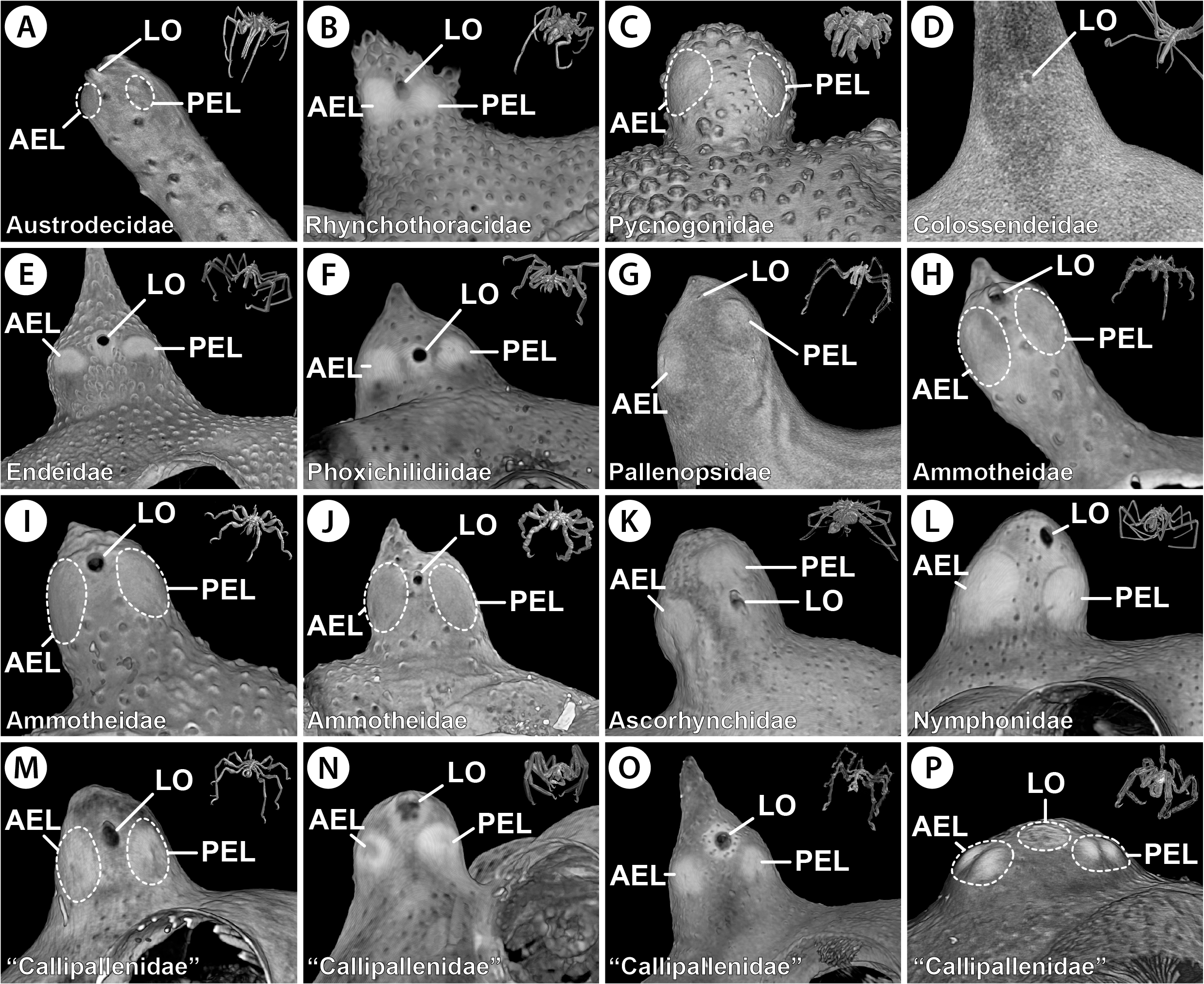

Supplement: Supplementary file 8 — Additional file 8: Figure S4: The eyes and lateral sense organ in all pycnogonid families. Volume renderings of the ocular tubercle, μCT scans in lateral view. If the eye lenses or lateral sense organs where not clearly discernible by external inspection alone, their presence was additionally confirmed by the study of the internal anatomy in the scans. A: Austrodecus glaciale (Austrodecidae). B: Rhynchothorax australis (Rhynchothoracidae). C: Pycnogonum litorale (Pycnogonidae). Note absence of the lateral sense organ. D: Colossendeis angusta (Colossendeidae). Note absence of the eyes. E: Endeis spinosa (Endeidae). F: Phoxichilidium femoratum (Phoxichilidiidae). G: Pallenopsis cf. aulaeturcarum (Pallenopsidae). H: Achelia echinata (Ammotheidae). I: Ammothea longipes (Ammotheidae). J: Tanystylum orbiculare (Ammotheidae). K: Ascorhynchus ramipes (Ascorhynchidae). Note upward shift of the posterior eye and the unusual position of the lateral sense organ below the latter. L: Nymphon gracile (Nymphonidae). M: Callipallene tiberii (“Callipallenidae”). N: Pallenella sp. (“Callipallenidae”). O: Parapallene avida (“Callipallenidae”). P: Stylopallene cheilorhynchus (“Callipallenidae”). Note external subdivision of the eye lenses. Abbreviations: AEL – anterior eye lens; LO – lateral sense organ; PEL – posterior eye lens. [file 12915_2021_1212_MOESM8_ESM.tif]

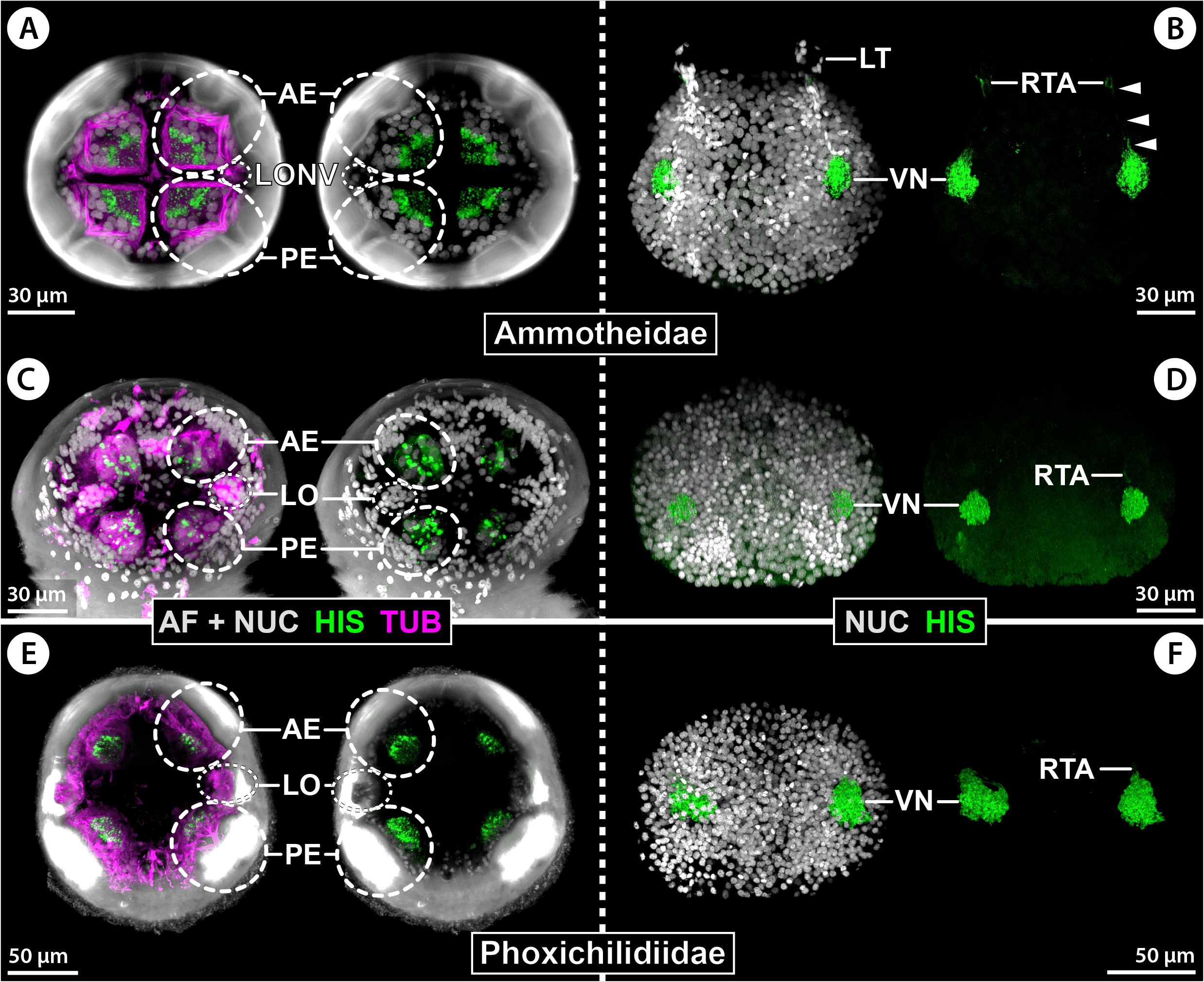

Supplement: Supplementary file 9 — Additional file 9: Figure S5: Histamine expression in eyes and first-order visual neuropil of Ammotheidae and Phoxichilidiidae. Extended optical sections of immunolabeled samples (MIP). A,C,E: Tubulin (TUB, magenta; left images only) and histamine (HIS, green) with nuclear counterstain and autofluorescence (AF + NUC, gray), horizontal sections through the ocular tubercle. Note absence of histamine labeling in the lateral sense organ. B,D,F: Histamine (green) with nuclear counterstain (gray, left images only), horizontal sections through the anterior protocerebral region. Note absence of histamine labeling in the median area housing the antero-median neuropil. A,B: Achelia echinata (Ammotheidae). C,D: Tanystylum orbiculare (Ammotheidae). E,F: Phoxichilidium femoratum (Phoxichilidiidae). Abbreviations: AE – anterior eye; LO – lateral sense organ; LONV – lateral sense organ nerve; LT – lateral thickening; PE – posterior eye; RTA – R-cell axons; VN – visual neuropil. [file 12915_2021_1212_MOESM9_ESM.tif]

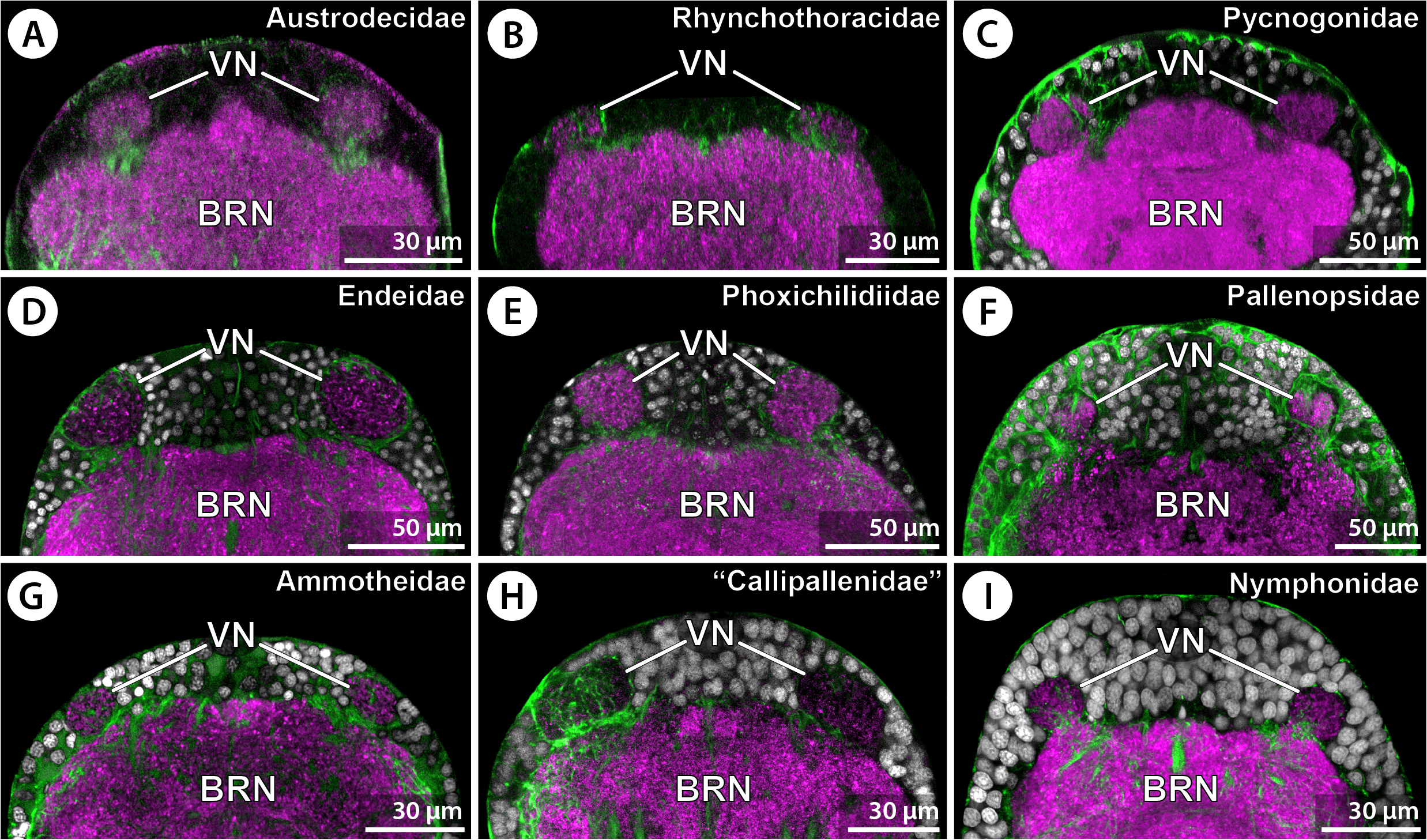

Supplement: Supplementary file 10 — Additional file 10: Figure S6: The first-order visual neuropil in various pycnogonid families. Tubulin (TUB, green) and synapsin (SYN, magenta) immunolabeling with nuclear counterstain (NUC, gray; except A and B), optical cross sections through the anterior protocerebral region. A: Austrodecus glaciale (Austrodecidae). B: Rhynchothorax australis (Rhynchothoracidae). C: Pycnogonum litorale (Pycnogonidae). D: Endeis spinosa (Endeidae). E: Phoxichilidium femoratum (Phoxichilidiidae). F: Pallenopsis sp. (Pallenopsidae). G: Achelia echinata (Ammotheidae). H: Callipallene brevirostris (“Callipallenidae”). I: Nymphon gracile (Nymphonidae). Abbreviations: BRN – brain neuropil; VN – visual neuropil. [file 12915_2021_1212_MOESM10_ESM.tif]

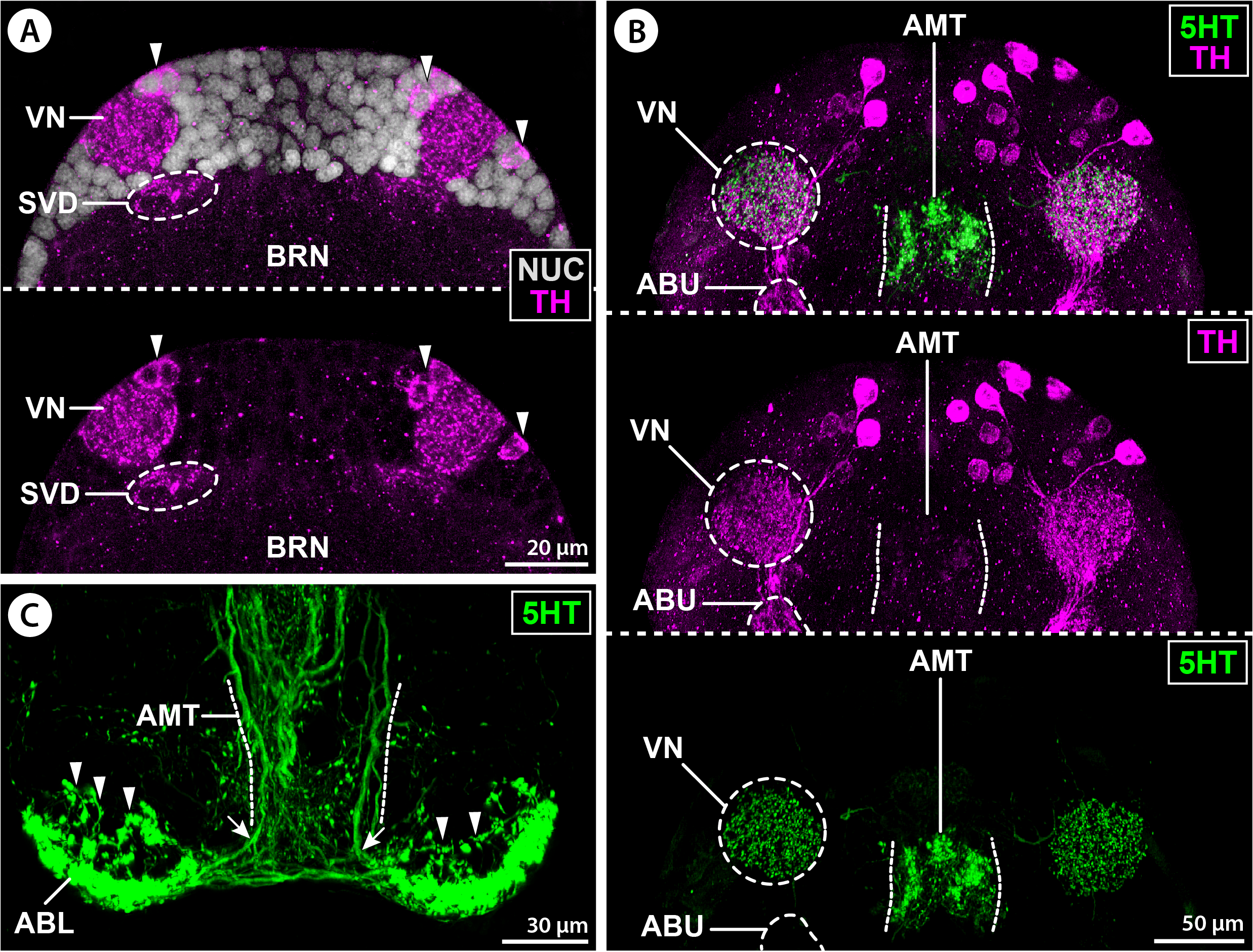

Supplement: Supplementary file 11 — Additional file 11: Figure S7: Selected details of protocerebral structures in Ammotheidae and Nymphonidae. Optical sections of immunolabeled samples (MIP). A: Achelia echinata, tyrosine hydroxylase (TH, magenta) with nuclear counterstain (NUC, gray; upper image only), cross section through anterior protocerebral region. TH-ir somata of type 1 interneurons (arrowheads) are located next to the TH-ir visual neuropil. Note also TH labeling in the sub-visual domain underlying the visual neuropil. B: Nymphon cf. multituberculatum, TH (magenta; upper and middle images) and serotonin (5HT, green; middle and lower images), extended horizontal section through anterior protocerebral region. Note TH and serotonin co-labeling in the visual neuropil. C: Nymphon gracile, serotonin, horizontal section through the antero-median tract and arcuate body. Serotonin-ir axons of ventral neurons project through the antero-median tract, bifurcate upon entry into the lower arcuate body layer (arrows) and form dense synaptic varicosities in its lateral arms. Note fine (and in part columnar) collaterals extending into the upper arcuate body layer (arrowheads). Abbreviations: ABL – lower arcuate body layer; ABU – upper arcuate body layer; AMT – antero-median tract; BRN – brain neuropil; SVD – sub-visual domain; VN – visual neuropil. [file 12915_2021_1212_MOESM11_ESM.tif]

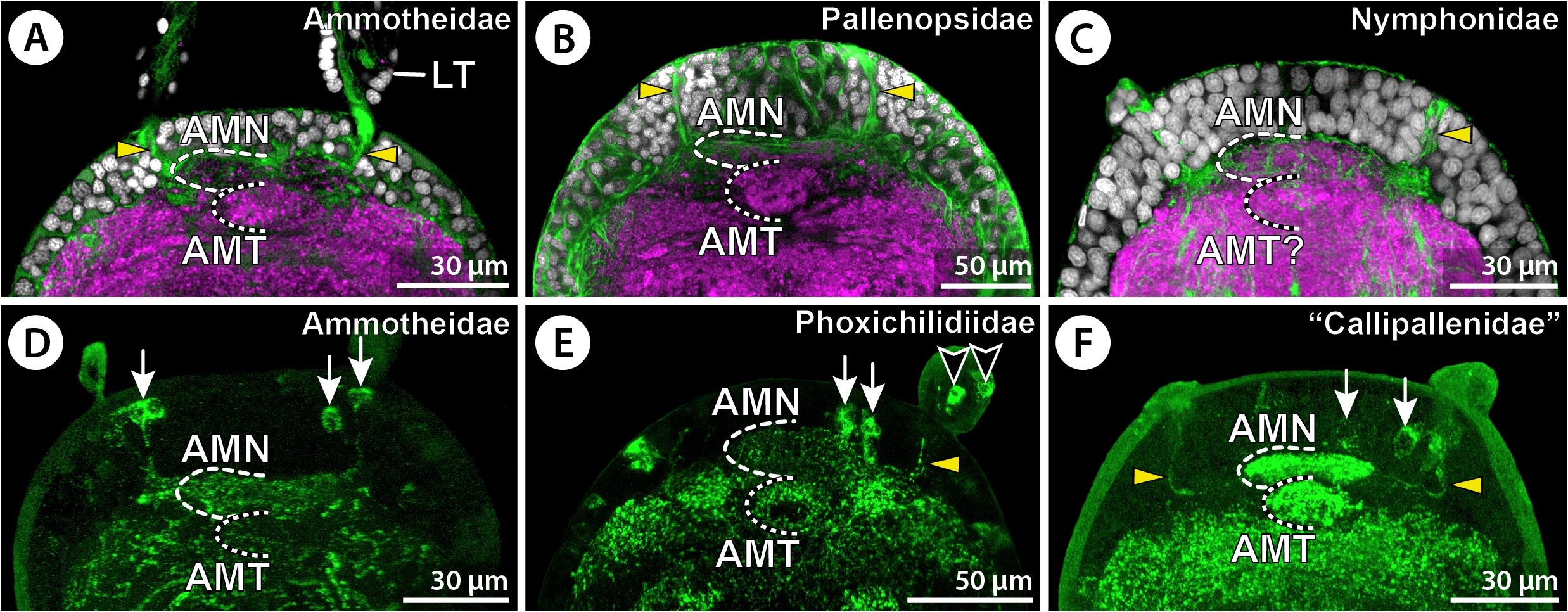

Supplement: Supplementary file 12 — Additional file 12: Figure S8: The antero-median neuropil and antero-median tract in various pycnogonid families. A-C: Tubulin (TUB, green) and synapsin (SYN, magenta) immunolabeling with nuclear counterstain (NUC, gray), optical cross sections through the anterior protocerebral region. Yellow arrowheads indicate the branch of the optic nerve looping toward the antero-median neuropil. A: Achelia echinata (Ammotheidae). B: Pallenopsis sp. (Pallenopsidae). C: Nymphon gracile (Nymphonidae). Note that the antero-median tract is not readily discernible in cross section, due to its rather diffuse, neuropil-rich nature in Nymphonidae (compare to Fig. 9E,F). D-F: Orcokinin (ORCO) immunolabeling, extended optical sections through the anterior protocerebral region (MIP). White arrows point to orcokinin-ir somata of anterior neurons that contribute neurites to the antero-median neuropil. Yellow arrowheads mark orcokinin-ir projections in the optic nerve branch extending to the antero-median neuropil. D: Tanystylum orbiculare (Ammotheidae). Note absence of distinct labeling in the antero-median tract. E: Phoxichilidium femoratum (Phoxichilidiidae). Note relatively weak labeling in the antero-median neuropil. Black arrowheads mark orcokinin-ir somata in the lateral thickening. F: Callipallene brevirostris (“Callipallenidae”). Note distinct orcokinin-ir projections (yellow arrowheads) from the optic nerve to the antero-median neuropil and strong signal in the antero-median neuropil as well as the antero-median tract. Abbreviations: AMN – antero-median neuropil; AMT – antero-median tract; BRN – brain neuropil; LT – lateral thickening. [file 12915_2021_1212_MOESM12_ESM.tif]

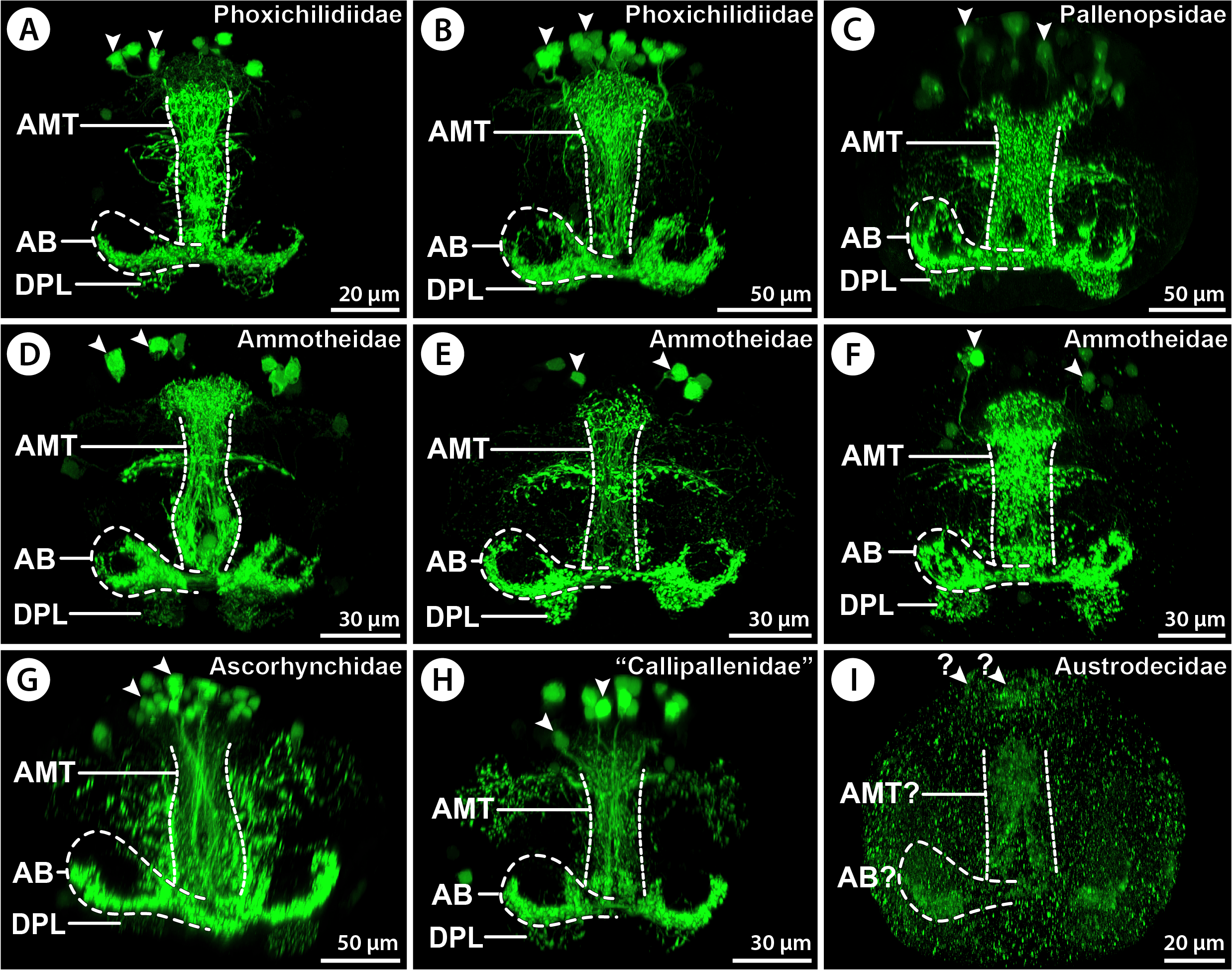

Supplement: Supplementary file 13 — Additional file 13: Figure S9: Serotonin expression in antero-median tract and arcuate body of various pycnogonid families. Serotonin (5HT) immunolabeling, extended horizontal sections through the brain (MIP). Arrowheads point to selected somata of ventral neurons that send projections along the antero-median tract into the arcuate body. A: Anoplodactylus pygmaeus (Phoxichilidiidae). B: Phoxichilidium femoratum (Phoxichilidiidae). C: Pallenopsis sp. (Pallenopsidae). Note relatively diffuse, neuropil-rich nature of the antero-median tract. D: Tanystylum orbiculare (Ammotheidae). E: Achelia echinata (Ammotheidae). F: Ammothella biunguiculata (Ammotheidae). Note relatively diffuse, neuropil-rich nature of the antero-median tract. G: Ascorhynchus auchenicus (Ascorhynchidae). H: Callipallene brevirostris (“Callipallenidae”). I: Austrodecus glaciale (Austrodecidae). Due to long-term PFA storage of the available material, the signal is extremely weak but still points to the presence of the serotonin-ir antero-median tract and lower arcuate body layer. Abbreviations: AB – arcuate body; AMT – antero-median tract; DPL – dorso-posterior lobe. [file 12915_2021_1212_MOESM13_ESM.tif]
